# Whither geographic proximity? Bypassing local R&D units in foreign university collaboration

**DOI:** 10.1057/s41267-021-00413-6

**Published:** 2021-04-12

**Authors:** René Belderbos, Marcelina Grabowska, Stijn Kelchtermans, Bart Leten, Jojo Jacob, Massimo Riccaboni

**Affiliations:** 1 Department of Management, Strategy and Innovation, Faculty of Economics and business, KU Leuven, Naamsestraat 69, 3000 Leuven, Belgium; 2 School of Business and Economics, Maastricht University, Tongersestraat 53, 6211LM Maastricht, the Netherlands; 3 UNU-MERIT, Boschstraat 24, 6211 AX Maastricht, the Netherlands; 4 Centre for R&D Monitoring ECOOM, Faculty of Economics and Business, KU Leuven, Naamsestraat 61, 3000 Leuven, Belgium; 5 Department of Management, Strategy and Innovation, Faculty of Economics and Business, KU Leuven, Warmoesberg 26, 1000 Brussels, Belgium; 6 Department of Management, Strategy and Innovation, Faculty of Economics and business, KU Leuven and University of Hasselt, Naamsestraat 69, 3000 Leuven, Belgium; 7 Department of Management, Technology and Strategy, Grenoble Ecole de Management, 12 rue Pierre Sémard, 38000 Grenoble, France; 8 IMT School for Advanced Studies, IMT Lucca, Piazza S. Francesco 19, 55100 Lucca, Italy

**Keywords:** industry–science linkages, research collaboration, foreign R&D, headquarters, geographic proximity, pharmaceutical and biotechnology industry

## Abstract

MNCs often engage in international research collaborations with foreign universities through one of their central R&D laboratories (at headquarters or elsewhere) even though they operate a local R&D unit close to that university, and hence forego the benefits of geographic proximity and local collaboration. Drawing on the knowledge-based theory of the firm, we hypothesize that the choice between distant and local collaboration systematically relates to the knowledge capabilities of the firms’ R&D units, the characteristics of the focal knowledge, and local knowledge leakage risks. Analysis of close to 13,000 research collaborations with foreign universities by the world’s major biopharmaceutical firms (1995–2015) confirms that collaboration at distance occurs if this allows the firm to benefit from scale and knowledge diversity advantages, if the central unit has strong basic research capabilities, and if collaboration is in a core research domain of the MNC while rival firms are locally present. Maturity of the focal research domain is associated with local collaboration. Our findings qualify the common arguments in favor of collaboration in proximity and suggest that (distant) central R&D units are important orchestrators of research collaboration with universities around the globe.

**Electronic supplementary material:**

The online version of this article (10.1057/s41267-021-00413-6) contains supplementary material, which is available to authorized users.

## Résumé

Les entreprises multinationales s'engagent souvent dans des collaborations de recherche internationales avec des universités étrangères par le biais de l'un de leurs laboratoires centraux de R&D (au siège ou ailleurs) même si elles exploitent une unité de R&D locale, proche de cette université. Elles renoncent ainsi aux avantages de la proximité géographique et de la collaboration locale. Nous appuyant sur la théorie de la firme fondée sur la connaissance, nous développons l’hypothèse que le choix entre les collaborations distantes et locales soit systématiquement lié aux capacités en matière de connaissances des unités de R&D des entreprises, aux caractéristiques des connaissances focales et aux risques de fuites de connaissances locales. L'analyse de près de 13000 collaborations de recherche des principales entreprises biopharmaceutiques (1995-2015) avec des universités étrangères confirme que la collaboration à distance se produira si cela permet à l'entreprise de bénéficier des avantages d'échelle et de diversité des connaissances, si l'unité centrale dispose de solides capacités de recherche fondamentale, et si la collaboration s’inscrit dans un domaine de recherche stratégique des multinationales alors que des entreprises concurrentes sont localement présentes. La maturité du domaine de recherche focal est associée à la collaboration locale. Nos résultats nuancent l'argumentation courante en faveur de la collaboration à proximité, et suggèrent que les unités centrales (et distantes) de R&D soient d'importants orchestrateurs de la collaboration de recherche avec les universités du monde entier.

## Resumen

Las empresas multinacionales con frecuencia participan en colaboraciones de investigación internacionales con universidad extranjeras mediante uno de sus laboratorios de I+D centrales (en la casa matriz o en otra parte) aún cuando operan una unidad de I+D local cerca a esa universidad, y de ahí que proceden los beneficios de la proximidad geográfica y la colaboración local. Basándonos en la teoría de la empresa basada en el conocimiento, planteamos la hipótesis de que la elección entre la colaboración a distancia y la local está sistemáticamente relacionada con las capacidades de conocimiento de las unidades de I+D de las empresas, las características del conocimiento focal y los riesgos de fuga de conocimiento local. Una análisis de cerca de 13.000 colaboraciones de investigación con universidades extranjeras por las empresas biofarmacéuticas más importantes del mundo (1995-2015) confirma que la colaboración a distancia se produce si esto permite a la empresa beneficiarse de las ventajas de escala y de la diversidad de conocimientos, si la unidad central tiene fuertes capacidades de investigación básica, y si la colaboración se produce en un dominio de investigación central de la empresa multinacional mientras que las empresas rivales están presentes a nivel local. La madurez del ámbito de investigación central está asociada a la colaboración local. Nuestros hallazgos habilitan la argumentación común a favor de la colaboración en proximidad y sugieren que las unidades centrales de I+D (distantes) son importantes orquestadoras de la colaboración en investigación con las universidades de todo el mundo.

## Resumo

MNCs frequentemente se envolvem em colaborações de pesquisa internacionais com universidades estrangeiras por meio de um de seus laboratórios centrais de R&D (na sede ou em outro lugar), embora operem uma unidade local de R&D perto dessa universidade e, portanto, renunciam aos benefícios da proximidade geográfica e colaboração local. Com base na teoria baseada no conhecimento da firma, formulamos a hipótese de que a escolha entre colaboração distante e local se relaciona sistematicamente com as capacidades de conhecimento das unidades de R&D das empresas, as características do conhecimento focal e os riscos locais de vazamento de conhecimento. Análise de cerca de 13.000 colaborações de pesquisa com universidades estrangeiras pelas principais empresas biofarmacêuticas do mundo (1995-2015) confirma que a colaboração à distância ocorre se isso permite que a firma se beneficie de vantagens de escala e diversidade de conhecimento, se a unidade central tiver fortes capacidades básicas de pesquisa , e se a colaboração está em um domínio de pesquisa central da MNC enquanto empresas rivais estão localmente presentes. A maturidade do domínio de pesquisa focal está associada à colaboração local. Nossas descobertas qualificam a argumentação comum em favor da colaboração na proximidade e sugerem que as unidades centrais (distantes) de R&D são importantes orquestradoras da colaboração em pesquisa com universidades ao redor do mundo.

## 概要

跨国公司(MNC)经常通过其中央研发实验室中的一个 (在总部或其它地方) 来与外国大学开展国际研究合作, 即使它们在该大学附近设有本地研发机构, 也会放弃地理位置接近和本地合作的益处。借鉴企业知识基础观, 我们假设在远程合作与本地合作之间进行的选择与企业研发机构的知识能力、焦点知识的特征以及本地知识泄漏风险有关。对世界主要生物制药公司与国外大学的近13,000项研究合作进行的分析 (1995-2015年) 证实, 远程合作在如下情况下发生: 如果这可以使公司从规模和知识多样化的优势中受益、如果中央机构具有强大的基础研究能力, 以及如果进行的合作是该MNC的核心研究领域而竞争公司在本地存在。焦点研究领域的成熟度与本地合作有关。我们的发现证实了支持近距离合作的普遍论点, 并表明 (远程) 中央研发机构是与全球大学开展研究合作的重要协调者。

## INTRODUCTION

The literature on the internationalization of R&D in multinational corporations (MNCs) has documented the importance of foreign R&D affiliates for firm innovation, with a more outspoken role in knowledge sourcing and creation (e.g., Song & Shin, 2008; Penner-Hahn & Shaver, 2005; Lahiri, 2010; Castellani et al., 2013; Belderbos, 2003; Kafouros et al. 2012; Manolopoulus et al., 2011; Belderbos et al., 2015). Foreign R&D units are given broader R&D mandates (e.g., Blomkvist et al. 2011, 2017; Cantwell & Janne, 1999; Cantwell & Mudambi, 2005) and some central R&D laboratories abroad assume global leadership in specific research domains, in line with a broader trend towards a more heterarchical organization of MNC competences and responsibilities (e.g., Frost et al., 2002; Menz et al. 2015; Nell et al., 2017). In our data on major firms in the global biopharmaceutical industry, we observe a clear trend toward a greater role of firms’ major R&D laboratories abroad, which in the most recent years in the observation period (2011–2015) have been responsible for more than a third of the main laboratory activities in scientific research.

An important role of foreign R&D units is to tap into local scientific networks in the host regions (Almeida & Phene, 2004; Ghoshal & Bartlett, 1990; Nohria & Ghoshal, 1997; Song & Shin, 2008). This follows from the notions that science is an important input for innovation in firms (Nelson, 1993; Cohen et al., 2002; Mansfield, 1995; Salter & Martin, 2001; McMillan et al., 2000; Bercovitz & Feldman, 2007), and that research collaboration with university scientists can improve firm performance (Belderbos et al., 2004; Cockburn & Henderson, 1998; Furman & MacGarvie, 2009). A key argument in the literature on university–industry collaborations is that these benefit from close geographic proximity (Abramovsky & Simpson, 2011; Belderbos et al., 2014; Boschma, 2005; Fabrizio, 2009). Such proximity reduces barriers to direct, face-to-face interactions between collaborators (Laursen et al., 2011; von Hippel, 1994), facilitates the exchange of tacit knowledge (Arora & Gambardella, 1990; Cockburn & Henderson, 1998; Nonaka, 1994), improves the trust between partners (Bruneel et al., 2010) and helps to overcome the significant institutional differences between universities and firms (Boschma, 2005; Ponds et al., 2007).

However, despite these benefits of geographic proximity, multinational companies (MNCs) often collaborate with universities located abroad at a substantial geographic distance (McKelvey et al., 2003; Ponds et al., 2007; Hoekman et al., 2009; Adams et al., 2005). This is not only due to a lack of R&D facilities in the proximity of foreign universities and the cost of setting up such facilities, but it also appears as a deliberate choice to involve one of the central R&Ds units rather than local R&D establishments in such collaborations. Our interviews with R&D managers of pharmaceutical MNCs confirmed that university R&D collaborations require the approval of R&D managers of a central laboratory and that decisions are likely to be part of a broader framework and program in which priorities are set. Collaborations are formal and involve contracts, which partially explains the systematic treatment and managerial involvement (e.g., Pisano et al., 2014).^1^ In general, (case study) evidence suggests a strong involvement of top management of MNCs in R&D decisions through the use of global R&D committees that gather, analyze, and approve plans of local units R&D and develop global R&D projects across units (Ivarsson et al., 2017; Belderbos, Lokshin, Boone & Jacob, 2020).

Our own data on the world’s leading biopharmaceutical companies reveal that in recent years more than two-thirds of MNCs’ research collaborations with foreign universities is carried out at distance by a central R&D unit (often at headquarters) even though the firms operate R&D facilities in proximity to the university partner. This share has been increasing, rather than decreasing, over the period 1995–2015 (see Table 2). Whether collaboration is local or with a central R&D unit elsewhere, moreover, often differs across collaboration projects (of the same firm) and varies with research domains and the local university context. For instance, AstraZeneca collaborated with the University of Sydney on asthma research in 2000 through its local R&D unit in New South Wales, and Sanofi Pasteur, the vaccines division of Sanofi-Aventis (headquartered in France) collaborated on penicillin research in 2014 with Harvard university via its US-based affiliate in Cambridge (MA). Conversely, Abbott partnered with the University of Oxford and Imperial College London on AIDS research in 2001 through its laboratory at headquarters in Illinois, bypassing its R&D unit near London. Similarly, Pfizer co-published research in the domain of organic chemistry in 2015 with the University of Cambridge (UK) carried out through its main lab in Connecticut (USA) rather than its local R&D establishments in the UK.^2^

The aim of this paper is to understand this phenomenon by systematically examining the conditions under which MNCs forego the potential benefits of local collaboration and rely on a central R&D unit elsewhere to collaborate with foreign universities at a distance. We suggest that there are important tradeoffs associated with organizing international research collaborations either through a central R&D unit or through a local R&D affiliate, which hitherto have not been given due attention. Drawing on the knowledge-based theory of the firm (e.g., Almeida et al., 2002; Foss et al., 2013; Grant, 1996), we identify a set of factors related to knowledge capabilities of the firm’s R&D units and characteristics of the knowledge domain that shape the organization of international research collaborations of the MNC. We distinguish between three features: the presence of cumulative knowledge in R&D units and the related knowledge creation, transfer and recombination capabilities of these units, the degree to which knowledge of the focal collaboration is novel, and the presence of knowledge spillover and misappropriation risks.

We analyze the decision to collaborate locally or via a distant central R&D unit in a sample of close to 13,000 research collaborations with foreign universities (as evidenced by scientific co-publications) by 49 major biopharmaceutical firms based in the United States, Europe, and Japan in 1995–2015.^3^ We find that a collaboration with a foreign university is more likely to be organized via a distant central R&D unit if (1) the MNC by doing so can benefit from the substantial scale and knowledge diversity benefits in the research domain of the collaboration, (2) the collaboration involves basic rather than applied research and the distant central laboratory has strong basic research capabilities, (3) the collaboration is not in a novel but in a mature research domain, and (4) the collaboration focuses on the core knowledge domains of the firm, in particular when a presence of local rivals increases the risk of dissipation and misappropriation of proprietary knowledge.

By addressing these issues, our paper qualifies the role of proximity in international R&D and contributes new insights to the literature on the internationalization of R&D in MNCs (e.g., Castellani & Lavoratori, 2020; Papanatasiou et al., 2019; Song & Shin, 2008; Penner-Hahn & Shaver, 2005; Belderbos et al., 2013; Cantwell & Mudambi, 2005; Lahiri, 2010; Castellani et al., 2013; Belderbos, 2003). Specifically, we respond to the call of a recent review on the internationalization of R&D (Papanatasiou et al., 2019) to take a broader, multidisciplinary perspective, by drawing on, and integrating, notions from the literature on R&D organization (e.g., Argyres & Silverman, 2004; Arora et al., 2011, 2014; Henderson & Cockburn, 1996; Leiponen & Helfat, 2011) and industry–science linkages (Bercovitz & Feldman, 2007; Bruneel et al., 2010; Laursen et al. 2011; Subramanian et al., 2013). Our theory focuses on the potential advantages of conducting R&D collaboration through central laboratories, and offers a novel perspective explaining this phenomenon. We adopt an inclusive perspective in which centralization decisions can relate to organizing university collaboration through the central laboratory at home but also through a central laboratory abroad with global authority in the domain. In contrast, existing literature has focused on the advantages of local collaboration through collocated R&D units referring to the greater possibilities of tacit knowledge exchange in proximity. Our analysis controls for these proximity benefits and provides evidence on the extent of the tradeoffs between centralization and such localization benefits.

## BACKGROUND AND HYPOTHESES

We begin with a review of the key literature relevant for our research, highlighting their main insights and the critical gaps they leave behind. Thereafter we propose a theoretical framework, building on the knowledge-based view of the firm, from which we subsequently develop our hypotheses.

The literature on *internationalization of R&D* has highlighted the important role that foreign subsidiaries play in the increasing internationalization of R&D activities by MNCs (e.g., Birkinshaw & Hood, 1998; Ghoshal & Bartlett, 1986; Pearce, 1989). This literature has pointed out that a significant source of the competitive advantage of MNCs is their ability to effectively coordinate and leverage innovative capabilities across the globe through their geographically dispersed network of R&D units (Foss, 2007; Faems et al., 2020). While MNCs have traditionally relied on foreign subsidiaries to exploit home-grown technologies by adapting them to local market needs (Foss et al., 2013; Kuemmerle, 1999), increasingly foreign R&D units operate with a knowledge-seeking and a knowledge-creation mandate (Florida, 1997; Frost, 2001; Frost et al., 2002; Gupta & Govindarajan, 2000; Nobel & Birkinshaw, 1998).

Subsidiaries contribute to the competitiveness of MNCs by tapping into local external networks in their host regions consisting of diverse actors, among which universities play a prominent role (Almeida & Phene, 2004; Cantwell, 2002; Ghoshal & Bartlett, 1990; Nohria & Ghoshal, 1997). Spatial proximity to foreign universities enables affiliate R&D units to tap into local scientific networks in the host regions (Almeida & Phene, 2004; Ghoshal & Bartlett, 1990; Nohria & Ghoshal, 1997; Song & Shin, 2008). Subsidiaries’ external networks can thus constitute a key strategic resource (Almeida & Phene, 2004; Cantwell & Mudambi, 2005; Frost, 2001; Song & Shin, 2008) as interactions within those networks expose subsidiaries to novel knowledge, ideas, and innovation opportunities not directly available in the home country (McEvily & Zaheer, 1999; Andersson et al., 2002; Anselin et al., 1997, 2000; Cooke, 2001; Leten et al., 2014; Faems et al., 2020; Asmussen et al., 2013). The presence of R&D subsidiaries in host regions, and their interactions with local actors, however, does not only provide firms with access to novel knowledge but it also entails a risk that knowledge dissipates to other actors in the region, including rival firms (Alcacer & Chung, 2007; Alcacer & Zhao, 2012; Belderbos et al., 2008).

Emphasizing the many advantages of geographic proximity, studies on *industry–science linkages* have suggested that firms tend to show a preference to locate R&D labs in close proximity of university research departments (Audretsch et al., 2005; Abramovsky & Simpson, 2011; Belderbos et al., 2014, 2017) and are more likely to collaborate with, or source knowledge from, local universities (Bruneel et al., 2010; Laursen et al., 2011; Arundel & Geuna, 2004; Belenzon & Schankerman, 2013). Spatial proximity to universities provides a substantial advantage to firms that want to keep abreast of recent scientific developments as spatial proximity facilitates the exchange of tacit knowledge and improves trust-building between firms and universities (e.g., Anselin et al., 1997, 2000; Cooke, 2001; Leten et al., 2014). Besides the positive performance effects of firm-university collaboration (Belderbos et al., 2004; Furman & MacGarvie, 2009; Du et al., 2014), there is evidence that proximate collaboration with universities can speed up firms’ innovation processes (Fabrizio, 2009). Prior studies (Fabrizio, 2009; Lim, 2004; Arora et al., 2018) have also highlighted the importance for firms of (collaborating on) *basic* scientific research and have called for a deeper understanding of how the phenomenon affects the productivity of firms’ innovation activities.

In contrast, the literature on the *organization of R&D* has emphasized the advantages of centralizing the R&D function in a single location. Centralization allows firms to benefit from economies of scale in specialized and indivisible human and physical resources (Poppo, 2003), and to reap economies of scope in large R&D laboratories where R&D activities span a diverse set of complementary technology fields (Argyres & Silverman, 2004; Argyres et al., 2020; Arora et al., 2011; Henderson & Cockburn, 1996; Leten et al., 2007). Furthermore, it has been argued that central R&D units conduct research that is more fundamental and scientific in orientation (Arora et al., 2011), and that the collocation of central R&D and corporate IP units offers advantages in terms of stronger control of knowledge (leakages) and more effective appropriation strategies (Collis et al., 2007; Di Minin & Bianchi, 2011).

The studies reviewed above, owing to their focus on a specific phenomenon (either local R&D or centralized R&D), have not considered the tradeoffs associated with a local (decentralized) or distant (centralized) organization of international research collaborations with universities. While a central tenet of the literature on international R&D and industry–science linkages is that research collaborations benefit from geographic proximity, a more comprehensive review of theoretical arguments highlights that there will be countervailing forces at play. Our theory focuses on such centralization advantages. Drawing on the knowledge-based theory of the firm, we present a theoretical framework that addresses this omission in prior literature and allows us to predict a firm’s choice between a central R&D unit at distance and a proximate affiliate R&D unit in organizing its international research collaboration with a foreign university.

### Theory and Hypotheses

The main premise of the knowledge-based view (e.g., Almeida et al., 2002; Foss et al., 2013; Grant, 1996) is that firms’ competitiveness rests to an important extent on their capacity to create, source, recombine, and exploit knowledge. It sees knowledge as the primary factor underlying firms’ competitive advantage, and firms’ cross-border knowledge transfer and recombination capabilities as crucial to the performance of MNCs (Kogut & Zander, 1992 & 1993). The knowledge-based view of the firm also holds that an appropriate organizational design is an important prerequisite for effective knowledge sourcing and recombination (Foss et al., 2013; Grant, 1996). It therefore provides a unified framework to understand the tradeoffs that firms confront in relation to local decentralized versus distant and centralized research collaborations with foreign universities.

The knowledge-based view sees university collaboration as providing exposure to (scientific) knowledge resources in a different organizational context which allows the MNC to keep abreast of a variety of research settings outside the confines of existing search routines and organizational practices. The efficient organization of this collaboration in the context of geographically dispersed R&D of the MNC is affected by a number of key parameters related to the characteristics of the focal knowledge and the knowledge capabilities of the R&D units of the firm. We distinguish three factors: the presence of cumulative knowledge and recombination and transfer capabilities in R&D units, the degree to which knowledge embeds tacit components or is unfamiliar to the firm favoring face-to-face interactions, and the perceived risks of knowledge spillovers to rival firms threatening effective appropriation of knowledge. As these features differ critically across firms and research domains, we can expect systematic differences in the propensity of university research collaborations to involve local R&D units or distant central R&D laboratories.

#### Cumulative knowledge: scale, scope, and basic research

We identify a potential role of cumulative knowledge in central R&D units related to processes of knowledge creation, transfer and recombination. In the knowledge-based theory of the firm, a notable feature of the knowledge creation process is the preference for firms to make use of new knowledge that is related to their accumulated repertoire of knowledge (Grant, 1996; Regnér & Zander, 2014). Knowledge development within firms is therefore a cumulative, path-dependent process, with firms’ combinative skills tied closely with their unique histories and the associated endowments of skills and knowledge (Teece et al., 1997). This suggests that the direction in which firms may advance their knowledge is influenced by the nature of their current knowledge (Leten et al., 2016; Stuart & Podolny, 1996). A key implication for the organization of a firm’s international research collaborations is that the central R&D unit may offer advantages in collaborations in research domains where the firm has historically accumulated substantial expertise and has reached a critical scale of knowledge creation. Although in the past a dominant argument in favor of decentralizing R&D activities was eliminating the difficulties of coordinating research efforts in multiple locations from a central unit, in recent years firms have been able to mitigate these challenges thanks to the advancements in information and communication technologies (Castellani & Lavoratori, 2020; Gray et al., 2015).

This ties in with the notion that R&D activities are typically characterized by strong scale economies, due to the presence of indivisible resources, such as laboratory equipment and specialized human capital (Kuemmerle, 1998). Empirical evidence on the organization of R&D has suggested that firms can benefit from conducting R&D in fewer locations, allowing for greater centralization (Argyres & Silverman, 2004; Argyres et al., 2020; Belderbos et al., 2013; Chacar & Lieberman, 2003). Centralization of R&D efforts enables firms to spread the high cost of specialized equipment and human capital and to reach the critical mass that is needed to efficiently utilize these resources (Poppo, 2003).

A larger centralized R&D unit also benefits from uninterrupted financial support even in times of economic downturns, which combined with its superior facilities and human capital, may give it an advantage in hiring top talent and attracting and hosting academic partners (Tirpak et al., 2006). In contrast, given that collaborative research in science rests heavily on scientists’ social networks (Zucker et al., 1998), a local unit with a lesser track record of publications in a domain will have fewer established academic relationships, hampering its legitimacy in local knowledge networks (Almeida & Phene, 2004; Andersson et al., 2002; Asmussen et al., 2013; Frost, 2001). This will put it at a less favorable position compared with its more high-profile central counterpart in establishing and executing university collaborations.

In summary, although local units possess some important advantages related to searching for and sensing new knowledge due to their greater ability for close interaction with local actors, a central R&D laboratory at distance with superior cumulative expertise can generate greater benefits related to knowledge creation by tapping into its scale and resource advantages. This is particularly so if the scale advantages of the central unit relative to the local unit are larger. This leads to the following hypothesis:

##### Hypothesis 1:

The likelihood that an MNC collaborates with a foreign university through a central R&D unit at distance (rather than through its local R&D unit) increases, the greater the scale of research capabilities in the central R&D unit vis-à-vis the local R&D unit in the research domain of the collaboration.

Not only knowledge creation, but also the processes of knowledge transfer and recombination have implications for the organization of international research collaborations with universities. In the knowledge-based view, processes related to knowledge transfer and recombination are perhaps even more important than those of knowledge creation and acquisition (Grant, 1996; Kogut & Zander, 1992 & 1993). As we discuss below, the efficient running of these two processes may warrant a centralized approach to international university collaborations, in particular if the central unit has accumulated capabilities in a variety of fields (knowledge diversity, Hypothesis 2), or in basic scientific research when the collaboration focuses on such basic research (Hypothesis 3).

Efficiently transferring and recombining knowledge among individuals and groups within a firm is in the knowledge-based view one of the fundamental capabilities of a firm. This is a critical function because the internal diffusion of knowledge is neither automatic nor easy, owing to the tacit nature of knowledge and the different ‘professional languages’ that exist within a firm. A firm is able to effect efficient internal diffusion and recombination of knowledge because it possesses the critical skill of translating knowledge elements from within, and also from outside, into a common language (Nonaka, 1994). This represents a unique, hard-to-imitate capability of the firm, defined as a ‘higher-order organizing principle’ (Kogut & Zander, 1992). Firms that have built up cumulative capabilities in multiple research domains are uniquely positioned to benefit from knowledge recombination by realizing knowledge spillovers across domains and developing innovations that combine different domains (Argyres & Silverman, 2004; Belderbos et al., 2013; Henderson & Cockburn, 1996; Leten et al., 2007). These benefits are also termed scope economies in R&D (e.g., Henderson & Cockburn, 1996).

It is primarily in central R&D laboratories with a broad knowledge base that knowledge developed and accumulated in one research domain can be efficiently transferred to and recombined with other domains (Argyres & Silverman, 2004; Argyres et al., 2020). Centralization provides firms with the flexibility to more easily and efficiently respond to the specific needs arising in a given collaboration by leveraging the central unit’s diverse cumulative pool of knowledge, technological know-how and infrastructure (Argyres & Silverman, 2004). Centralization may also help to enhance the innovation potential of the joint research with the university partner because of the close links of diversified central laboratories with other parts of the organization, such that diverse research outcomes may find more applications, in particular those that match better with the needs of the firm. This increases the recombination potential of knowledge created in the collaboration, and the transition into the later stages of development and commercialization (Kogut & Zander, 1993; Ketokivi & Ali-Yrkko, 2009). R&D managers of central laboratories are also incentivized to create such firm-wide synergies and to cater to the innovation needs of the firm as a whole (Coombs & Richards, 1993; Lerner & Wulf, 2007). Recent evidence suggests that with centralization of R&D authority, internal R&D collaboration is enhanced (Argyres et al., 2020).

Local R&D units may on balance hold little proximity-related advantages because the transfer and integration of external knowledge distance to other units of the firm may matter as much as the distance to the collaboration partner (Papanatasiou et al., 2019). Collaborative research through decentralized units may even be structurally inconsistent with the processes of knowledge transfer and recombination because, to the extent that local units are rewarded based on their individual performance, they would have limited incentive to transfer knowledge to other units or to engage in joint research with these units (Bercovitz & Feldman, 2007; Leiponen & Helfat, 2011). Furthermore, a local R&D unit that is geographically distant from the headquarters is less embedded within the firm and therefore less able to get the attention of headquarters to effectively transfer the knowledge it acquires to the relevant parts of the firm (Bouquet & Birkinshaw, 2017).

These arguments suggest that the greater the opportunities are for reaping knowledge diversity and recombination advantages in a distant central R&D unit, the greater the benefits of collaborating with a foreign university through this R&D unit rather than through the local R&D unit. This leads to the following hypothesis:

##### Hypothesis 2:

The likelihood that an MNC collaborates with a foreign university through a central R&D unit at distance (rather than through its local R&D unit) increases, the greater the knowledge diversity of the central R&D unit vis-à-vis the local R&D unit.

Comparable arguments on the roles of intra-firm knowledge transfer and knowledge recombination apply when considering a core distinction in academic research: the difference between basic and applied research. Basic research addresses fundamental questions, aims at a greater understanding of important phenomena, and is not guided by specific practical needs (Nelson, 1959; Pavitt, 1991; Salter & Martin, 2001). Basic scientific knowledge has important advantages for innovating firms (Arora et al., 2018; Fleming & Sorenson, 2004; Rosenberg, 1990) and firms seek to collaborate with university scientists to secure access to such knowledge (Tapon & Thong, 1999; Fabrizio, 2009). The nature of basic scientific knowledge focusing on fundamental insights is likely to be useful for a variety of applied research efforts, in multiple – in the context of biopharmaceutical research – therapeutic fields and drug development programs. Studies note the importance that central R&D units attach to fundamental research and its positive contribution to overall firm performance (Arora et al., 2011; Chandler, 1991; Foss, 1997; Goold et al., 2001).

First, central R&D units are often charged with internal knowledge transfer and diffusion of knowledge within the MNC network (Chandler, 1991; Foss, 1997; Gupta & Govindarajan, 2000; Ciabuschi & Martin, 2010; Awate et al., 2015). Given the potential company-wide benefits of basic research (Lerner & Wulf, 2007; Della Malva et al., 2015; Argyres et al., 2020), a central R&D unit, especially when it has already built a superior basic research capability compared with a local unit, may therefore be best placed to collaborate with foreign universities in basic research and to diffuse the resulting knowledge across the different units and projects within the MNC’s network. A central R&D unit also commands greater corporate support and authority that is required for coordinating the integration of the typically complex knowledge emanating from basic research (Christensen, 2002). Engaging in basic research hence requires a degree of decision power in relation to changing the direction of research and accepting research outcomes that may not readily have applications within the firm. Local affiliates are less likely to meet these criteria because they have relatively lower decision autonomy and may have mandates for more specific research outcomes (Ambos & Ambos, 2011; Bercovitz & Feldman, 2007; Blomkvist et al. 2011, 2017). Local R&D units may also be less aware of the R&D activities and knowledge requirements of other MNC units (Egelhoff, 2010), and may be less willing to share knowledge due to reward systems that are mainly based on the performance of their own unit (Arora et al., 2011).

Second, conducting basic research through the central laboratory can enable top management to timely identify new opportunities and directions for the firm and hence ensure its long-term competitive advantage (Brown, 1991; Zahra et al., 2018). In contrast, when basic research is carried out through dispersed local R&D units, which are geographically distant and insufficiently incentivized to share knowledge, managers in higher echelons may have difficulties in developing a clear foresight about the long-term evolution of technologies and products, hampering their ability to affect organizational renewal and long-term growth (Aguilera et al., 2019; Bercovitz & Feldman, 2007; Lerner & Wulf, 2007).

Third, the highly uncertain nature of basic research with only long-term payoffs (Rosenberg, 1990) is likely to make it less attractive to pursue for managers of affiliate R&D units, which may lead to centralized design choices. Central R&D units’ organization and incentive structures make them better geared to support longer-term, ‘context-transcending’ research with potential applications in a wide range of businesses (Galunic & Eisenhardt, 2001; Lerner & Wulf, 2007). R&D managers at central R&D units tend to face less stringent time constraints and market pressures, allowing them greater freedom to engage in collaborative basic research projects with foreign universities that have a broad focus and are not directly geared to addressing immediate or specific customer needs (Argyres & Silverman, 2004). Bercovitz and Feldman (2007) note in this regard that firms with more centralized R&D structures commit more R&D resources to university research.

In sum, the centralized organization of basic research collaboration with universities benefits from the central unit’s basic research capabilities combined with its long-term orientation, its authority and incentives to coordinate knowledge integration, its understanding of the knowledge bases and knowledge needs of the company, and its influence on the strategic decisions of the company. These advantages of collaboration in basic research through a central R&D unit are more pertinent the greater the cumulative basic research capabilities of the central laboratory in comparison with the local R&D unit. We hypothesize:

##### Hypothesis 3:

The likelihood that an MNC collaborates with a foreign university through a central R&D unit at distance (rather than through its local R&D unit) increases, the greater the scale of basic research capabilities in the central R&D unit vis-à-vis the local R&D unit in the research domain of the collaboration, provided that the collaboration involves basic research.

#### The nature of knowledge in collaborative research: novel vs. mature domains

The knowledge-based view of the firm regards knowledge recombination as an extremely difficult process, especially when the knowledge in question contains a substantial tacit component and if firms are less familiar with the knowledge (Kogut & Zander, 1992; Nonaka, 1994). Tacit knowledge is difficult to articulate and can only be acquired through observation and practice (Grant, 1996; Polanyi, 1966). This explains the frequent face-to-face interactions between individuals, as documented by numerous studies, taking place in geographically concentrated, close-knit networks of scientists and inventors around the world (Von Hippel, 1994; Saxenian, 1994). Hence, the benefits of geographic proximity will also depend on the nature of the knowledge developed in collaborative research. We argue that the nature of knowledge, in particular its tacitness, suggests implications for the role of research in novel (versus mature) research domains.

The advantage of foreign R&D subsidiaries of MNCs embedded in local research networks has been related to their capacity to scan for and access new technologies and developments in science (Almeida & Phene, 2004, Song & Shin, 2008; McEvily & Zaheer, 1999; Andersson et al., 2002; Faems et al., 2018). Knowledge pertaining to novel technologies and new research domains tends to have a high tacit component that is embodied in those who generated new knowledge (Cohen & Levinthal, 1990; Polanyi, 1966) and is intricately bound to the context in which it has been developed (Ambrosini & Bowman, 2001; Lissoni, 2001; Nonaka, 1994).

Successful absorption of tacit knowledge requires dense communication channels (Szulanski, 1996) and an understanding of the context in which the knowledge is developed and employed (Kogut & Zander, 1993). The presence of an R&D unit in a host region allows tapping into the local scientific network, facilitating personnel movements and frequent face-to-face interactions with local collaborators to absorb such tacit knowledge (Laursen et al., 2011; Leten et al., 2014). In this respect, prior research highlights the supportive role of universities in the development of distinctive expertise in firms’ collocated R&D laboratories. Furman and MacGarvie (2009) describe the important contribution of universities in the emerging phase of the pharmaceutical industry through collaboration and training of scientific and technical staff. R&D units co-located and collaborating with universities on pioneering research and novel research domains can become part of the local social network of scientists typically associated with emerging knowledge hotspots and new developments in the biopharmaceutical industry (Liebeskind & Oliver, 1998; Owen-Smith & Powell, 2004; Zucker et al., 1998).

These considerations are less important if the collaborative research with foreign universities involves mature domains of research. Face-to-face interactions on a regular basis are less important to recombine knowledge in mature domains with established scientific principles, and the distinctive contribution of local foreign R&D units in knowledge sourcing will be less salient. Central R&D units are in this case at an advantageous position in leveraging their cumulative assets and expertise to combine insights from collaboration with the existing knowledge base, and to aim for cross-fertilization across collaborations and R&D projects. The above arguments suggest the following hypothesis:

##### Hypothesis 4:

The likelihood that an MNC collaborates with a foreign university through a central R&D unit at distance (rather than through its local R&D unit) increases in the maturity of the research domain involved.

#### Knowledge appropriation and knowledge spillover risks: core domains and local rivals

The knowledge-based view of the firm also emphasizes that an effective appropriation of knowledge is crucial for competitiveness. Appropriability of knowledge refers to the ability of the owner to generate an economic return equal to the value of the knowledge (Grant, 1996; Teece, 1986). The risk of knowledge dissipation to competitors may hamper the appropriation of knowledge. The nature of knowledge transfer processes often implies that the translation of knowledge residing in individuals into a common language through simplification and codification not only facilitates internal knowledge diffusion and recombination but also knowledge leakage and imitation (e.g., Winter, 1987; Kogut & Zander, 1992). When knowledge leaks out, competitors may free ride on the investments made by a firm at a comparably modest learning cost (Zucker et al., 1998) and misappropriate its knowledge. Firms that engage in research partnerships with foreign universities risk that sensitive knowledge and company secrets shared and developed in the joint research with the academic institution may leak out to other firms.

The risk of knowledge dissipation and the associated threat to the effective appropriation of the fruits of R&D efforts will be most salient if the collaboration takes place in research domains of core competence of the firm– in which the firm has built up valuable cumulative experience and proprietary knowledge. Firms’ core (scientific) research competencies can be viewed as a set of unique and idiosyncratic knowledge resources (Hamel, 1994) with strategic significance and critical to a firm’s competitive advantage. This set of critical knowledge resources – when deployed effectively – generates superior value for a firm, setting it apart from its competitors (Kogut & Zander, 1996). Consequently, these critical knowledge resources are central to, and constitute the basis for, sustainable competitive advantage (Hamel, 1994; Leonard-Barton, 1992). Defined as organization-specific arrangements and scientific expertise of employees (Polanyi, 1962), core scientific knowledge constitutes such a fundamental resource of a firm (Scott, 1998).

While the consequences of knowledge spillovers are more serious in core scientific domains, we argue that the likelihood that such knowledge spillovers occur differs depending on whether the collaboration is organized through the central or local R&D laboratory. There are two interrelated factors influencing the likelihood of spillovers: collaboration partners’ access to knowledge, and the firm’s control over knowledge. In terms of access, on the one hand, university scientists who collaborate with a foreign firm through its central R&D lab are exposed to a larger and wider knowledge base than they would be when working with a local laboratory. This broader inroad into the firm’s proprietary knowledge base increases the risk of spillovers to other actors in the university region, especially if the collaborating academics have extensive business networks (Aldridge & Audretsch, 2010; Grimaldi et al., 2011). On the other hand, collaborating through the central laboratory implies a lack of physical proximity between firm and university scientists, which reduces the possibilities for rich interaction that increase the likelihood of knowledge spillovers. Hence, although working with a local R&D unit offers a more modest foray into the firm’s knowledge base, this limited access is counterbalanced by the positive influence of physical proximity on knowledge transfer and spillovers. Proximity reduces barriers to direct, face-to-face interactions between collaborators (Teece, 1986; Polanyi, 1966; von Hippel, 1994; Lane and Lubatkin, 1998; Nooteboom, 2000; Laursen et al., 2011), facilitates the exchange of tacit knowledge (Arora & Gambardella, 1990; Jaffe et al., 1993; Nonaka, 1994; Cockburn & Henderson, 1998; Almeida & Kogut, 1999; Narula and Santangelo, 2009; Belenzon & Schankerman, 2013), improves trust between partners (Bruneel et al., 2010) and helps to overcome the significant institutional differences between universities and firms (Arrow, 1962; Boschma, 2005; Nelson, 1959; Ponds et al., 2007). On balance, therefore, it may be that effective access to knowledge is greater for local R&D unit collaboration in proximity than for distant collaboration with a central laboratory.

In terms of control and knowledge protection, organizing for foreign university collaboration through a central R&D unit is likely to have clear advantages. The often-available expertise on intellectual property management in central research units will allow for closer control and management of knowledge dissipation risks (Argyres & Silverman, 2004; Di Minin & Bianchi, 2011). Di Minin and Bianchi (2011) argue in this regard that R&D at central laboratories can constitute a “safe nest” for strategic R&D projects. Similarly, Alcacer and Zhao (2012) find that MNCs with a (foreign) R&D unit collocated with rival units are likely to make use of cross-border R&D teams involving a central R&D laboratory, and that this setup is associated with reduced knowledge spillovers. They argue that one of the purposes of the involvement of the central R&D laboratory is the greater control that can be exercised on the distribution and protection of the MNC’s knowledge.

Summarizing, while firms that collaborate with foreign universities through their distant central R&D unit may expose more knowledge than those who partner via a proximate local R&D unit, the greater physical distance and the available expertise on intellectual property management in central R&D units are expected to better protect firms against knowledge spillovers and misappropriation in university collaboration. These considerations are most prominent in core research domains, where the negative consequences of outgoing knowledge spillovers or the focal firm are strongest. The above arguments lead to the following hypothesis:

##### Hypothesis 5:

The likelihood that an MNC collaborates with a foreign university through a central R&D unit at distance (rather than through its local R&D unit) is greater if the collaboration involves a core research domain of the firm.

The consequences of knowledge leakages due to foreign university collaboration will be especially severe when knowledge dissipation is more likely to benefit direct competitors (Alcacer & Chung, 2007; Shaver & Flyer, 2000; Belderbos et al., 2008), i.e., if rival firms are active in the foreign region where the university is located. In that case, the advantages of centralization of collaborations at the distant central R&D unit in terms of retaining tighter control of firms’ proprietary knowledge resources will be even more critical (Alcacer & Zhao, 2012). This suggests that the benefits of organizing foreign research collaboration in core domains through a central R&D unit will be stronger the greater the number of collocated rivals operating in the foreign region of the university.

##### Hypothesis 6:

The effect of the involvement of core research domains in foreign university collaboration on the likelihood that an MNC collaborates through a central R&D unit at distance (Hypothesis 5) is stronger, the greater the number of rival firms present in the host region of the foreign university.

## DATA, VARIABLES, AND EMPIRICAL METHODS

### Sample and Data

We constructed a dataset on the research activities of 148 top R&D spending firms in the pharmaceutical industry, covering the period 1995–2015. The sample firms have headquarters in the United States, the EU, and Japan and have been selected as the top R&D spending (bio)pharmaceutical firms from the ‘2004 EU Industrial R&D Investment Scoreboard’ and a list of the largest patentees in biotechnology at the European Patent Office in the year 2005. The EU Industrial R&D Investment Scoreboard lists the top 500 corporate investors in R&D with the home base in the EU, and the top 500 companies with their home base outside the EU (mainly the US and Japan), based on corporate R&D expenditures in 2003.

Information on scientific publications in peer-reviewed international journals is used to identify the involvement of firms in (collaborative) research with universities. Prior work has argued that publication counts represent investment levels in science and is a proxy for the extent to which companies are involved in scientific research (Gambardella, 1992). In addition, publication rates are a timely measure of firms’ involvement in scientific research since the turn-around time of publications in most natural sciences is short (Kaplan et al., 2003).

Publication data are extracted from PubMed, the largest biomedical literature database in the world, and collected and consolidated at the firm level. This approach consists of identifying all publications on which the parent firms or their subsidiaries are listed as publishing institutes. We relied on ORBIS-Bureau Van Dijk for affiliate and consolidation information, in addition to lists of firms’ subsidiaries reported in corporate annual reports, yearly 10-K filings in the United States, and for Japanese firms, information on foreign subsidiaries published by Toyo Keizai in the yearly Directories of Japanese Overseas Investments. The consolidation was implemented on a yearly basis to account for changes in group structures over time. Acquired companies and their publications are considered part of a parent firm from the year of acquisition onwards.

One of the advantages of using PubMed is that the articles are indexed with Medical Subject Headings (MeSH). MeSH terms constitute a controlled vocabulary maintained by the National Library of Medicine that provides a fine-grained classification of biomedical research domains. Publications in the database are tagged with a set of MeSH keywords by professional indexers and not by the authors themselves. The MeSH classification consists of a hierarchical tree covering 16 separate branches that can reach up to 12 levels of depth. Given the aims of our research, we limited our analysis to articles assigned to the two main branches that are relevant to the process of biopharmaceutical innovation, i.e., “Diseases” (Category C) and “Drugs & Chemicals” (Category D), which cover about 96% of the publications of the sample firms. The MeSH classification is very fine-grained: the categories “Diseases” and “Drugs & Chemicals” contain 13264 different MeSH terms, such as “RNA virus infections” and “SMAD proteins”.

To identify the research domain(s) of a publication, we used MeSH subject headings at the second level of disaggregation (three-digit) of the MeSH classification (e.g., Bignami et al., 2019), with the exception of the calculation of maturity, for which we use all levels of the MeSH tree. Within the categories “Diseases” and “Drugs & Chemicals” there are 42 different three-digit MeSH terms, which typically list the type of disease (such as eye –, nervous system – or cardiovascular disease) and the type of biochemical matter involved (such as polycyclic compounds, organic chemicals).^4^ Most publications therefore list more than one of these keywords. Hereafter we refer to the three-digit MeSH keywords listed on a focal publication as the (combined) *research domain* of the publication.

### Research Collaborations with Foreign Universities

Firms’ research collaborations with universities are identified through co-publications, in line with prior research (e.g., Cockburn & Henderson, 1998; Fabrizio, 2009). Co-publications are considered a reliable indicator of research collaborations (Laudel, 2002). Hicks (1996) concluded that the large majority (84–93%) of co-publications of a sample of Japanese firms involved a collaboration of some sort. Conversely, research collaboration also typically leaves a ‘paper trail’ in the form of co-publications: Melin and Persson (1996) reported that only 5% of surveyed scientists indicated instances of collaboration not resulting in co-authored papers. In sum, most scientific collaborations result in co-authorship of publications, and most co-publications result from scientific collaborations.^5^ To identify the publications that are jointly published with universities, we coded the presence of the words *university*, *college,* and (local) variants in the co-authors’ affiliation names mentioned on firms’ publications.

To determine the geographic dimension of university–firm collaborations, we geocoded the affiliations on scientific publications using the address information provided (Catini et al., 2015). The geocoding of publications was implemented at the OECD Territorial grid two-level (OECD, 2018), covering NUTS one or two-level in European countries, (groups of) prefectures in Japan, and states for the US. The NUTS-2 level has been used in prior research linking regional R&D location choice of firms to the presence of universities (e.g., Belderbos et al., 2014) and reflects that collaboration frequently occurs within a broader area as long as a daily commute is possible. University–firm collaborations are considered as ‘foreign’ when the university is located outside the home country of the firm. The home country of the firm is defined as the country where the headquarters is located.

### Central R&D Units

We used information on the geography of the firms’ subsidiaries listed on firm publications to map the global R&D network of firms and to identify the firms’ central R&D laboratories and whether firms have a local R&D unit in the host region of the collaborating foreign university. We define central R&D units as those R&D locations that are responsible for the largest number of publications in a research domain, as our theory and research question focus on the tradeoff between local R&D collaboration in proximity and distant collaboration with a central laboratory that has scale and scope advantages. We apply a minimum size criterion of a total of 50 publications in the prior 4-year period to bring in consistency with the notion of a central laboratory. This threshold ensures that the central laboratory can easily be identified as the largest of the firm in a domain, with other laboratories in the domain substantially smaller.^6^ Inspection of the known locations of major R&D sites of some firms confirms that the publication-based measure can accurately identify central laboratories and their domain specialization. For instance, for GSK, we identify central laboratories in Belgium, Italy, the UK, and the US.^7^ The requirement to define central laboratories with a size criterion does imply the exclusion of observations on smaller biotech firms with limited publication activities in a domain for which no main R&D laboratory can be identified.^8^

Table 1 shows the trend in the number of central laboratories and their locations. The numbers focus on the 49 firms that are included in the sample for analysis (see below) in order to provide an accurate context. The average number of central laboratories is increasing over time but still only reaches 2.3 in the period 2011–2015, indicating that R&D activities remain concentrated in a limited number of laboratories. What is clearly visible is that the number and share of central laboratories outside the home country is increasing. The share of foreign central laboratories in all central laboratories increased steadily from 15% in 1995–2000 to close to 33% in 2011–2015. Although the averages over 49 firms are relatively low, there are several (larger) firms that operate a multitude of central laboratories, of which quite a few are located abroad, such as Novartis, Sanofi, Pfizer, GSK, AstraZeneca, Schering Plough, Boehringer Ingelheim, Johnson & Johnson, UCB, Eisai, Roche, and Takeda. On average, about half of the foreign laboratories are located in the US (laboratories of European and Japanese multinationals), with Sweden and the UK also hosting around 10% of the central laboratories. In terms of publication output, more than a third of the total publications of the central laboratories of the firms originated from foreign central laboratories in the most recent years (2011–2015), up from 13% in 1995–2000. Among domestic central laboratories, the large majority of publications is due to the central laboratory at headquarters.

**Table 1:** Foreign and domestic central laboratories, 1995–2015

	1995–2000	2001–2005	2006–2010	2011–2015
Average number of central R&D laboratories				
Total	1.7	2.0	2.2	2.3
Laboratory at headquarters	0.7	0.6	0.7	0.7
Laboratories elsewhere in home country	0.7	0.9	0.8	0.9
Laboratories abroad	0.3	0.4	0.6	0.7
*Share of laboratories abroad*	*0.15*	*0.21*	*0.30*	*0.31*
Number of publications of central R&D laboratories				
Total during the period	592.8	623.7	678.7	849.6
Average yearly publications:				
Laboratory at headquarters	297.2	277.5	289.5	375.3
Laboratories elsewhere in home country	230.4	235.8	221.9	231.7
Laboratories abroad	75.1	127.1	193.6	304.1
*Share of laboratories abroad*	*0.13*	*0.20*	*0.29*	*0.36*

Averages of the 49 firms included in the analysis of Table 4. Central laboratories are laboratories with the largest number of publications in a research domain, with a minimum of 50 publications in the prior 4-year period.

### Co-publication Trends and Sample for Estimation

Table 2 provides details on trends in the nature of foreign university collaboration over the period 1995–2015 of the 148 firms and shows how the sample for estimation is created. The first panel (Panel A) shows that the phenomenon of foreign university collaboration has been gaining importance. Among the 148 firms on which data were collected, 144 have at least one co-publication with a foreign university. The number of co-publications with foreign universities increased from 12,284 during 1995–2000 to 15,053 during 2011–2015. These numbers also represent a rising share of all publications of the firms, from 27% to more than 40%, respectively. Yet, despite the increase in foreign central laboratories, there has not been an increased role of collaboration in proximity. The share of foreign university collaboration that involves the local R&D unit in proximity of the university has been ss at about 17%. Hence, even with a greater role of central R&D laboratories located abroad, the role of R&D collaboration in proximity has not become more prominent.

**Table 2:** Co-publications with foreign universities: trends and sample selection

	1995–2000	2001–2005	2006–2010	2011–2015	Total	No. of firms
Panel A. Firms' co-publications with foreign universities						
Number of copublications during the period	12,284	14,668	13,587	15,053	55,592	144
As share of total firm publications(%)	27.5%	31.5%	34.7%	40.9%	33.2%	
Of which: collaboration through the local R&D unit (%)	17%	17.1%	16.7%	16.7%	16.9%	
Panel B: Firms' collaborations with foreign universities – with local R&D option	3115	4477	4533	6369	18494	61
As % of foreign university copublications (%)	25.4%	30.5%	33.4%	42.3%	33.3%	
Of which omitted:						
Local R&D unit is the main laboratory	541	457	660	1033	2691	
Joint collaboration with both local R&D unit and main	61	153	157	388	759	
Main laboratory is elsewhere in the host country	420	360	560	840	2180	
Firms with only one type of collaboration outcome	3	3	24	26	56	
Total sample observations local vs. distant collaboration	2090	3504	3132	4082	12,808	49
Of which: collaboration through the local R&D unit (%)	51.3%	45.2%	33.8%	28%	37.9%	

Numbers for 148 sample firms. Collaboration counts in panel B identify foreign university collaborations where a central laboratory can be identified in the research domain as distant alternative and if the firm has prior publication activity in proximity to the foreign university (a local R&D option).

In our empirical analysis, we focus on the phenomenon of R&D collaborations with foreign universities where there is a clear tradeoff and decision to make on local versus distant collaboration. In order to arrive at a measure of individual R&D collaborations derived from co-publications, three steps have been taken. First, our focal observations are on R&D collaborations, but an R&D collaboration can lead to multiple publications. We therefore omit ‘duplicate’ collaborations, which we define as a firm - foreign university co-publication in the same year and research domain.^9^ Second, the same co-publication can involve multiple universities located in the same region or in another region or country. We consider each university collaboration as a separate observation, which increases the number of observations at the collaboration level by about 16%. Third, and most substantive, we maintain only those collaborations in instances where the firm operated an R&D unit in proximity to the foreign university, and when the firm has a clear choice to either collaborate locally through the local R&D unit or internationally through a central R&D laboratory at distance. In the absence of such existing local R&D activity, the choice for a local collaboration would entail investments in setting up a new R&D unit in proximity to the university. While this is a theoretical possibility, the odds would be very much stacked against local collaboration and the local collaboration option may often not be seen as a valid one. Focusing on foreign university collaborations as defined above, with a local R&D option and a central laboratory as the alternative, in Panel B of Table 2 we see increasing numbers over time, from about 3115 during 1995–2000 to about 6369 during 2011–2015. The number of firms involved in such collaborations with relevant local versus distant collaboration options is reduced to 61. The collaborations with a local option represent an increasing share of foreign university co-publications, from 25% to about 42%. This attests to the increasing dispersion of R&D activities of MNCs in the biopharmaceutical industry.

Panel B of Table 2 also shows how we arrive at the precise sample for estimation. Four categories of foreign university collaboration are omitted from the analysis. First, there are cases where the local R&D option is the central laboratory, which implies there is no tradeoff on distant versus local collaboration and our theory and empirical model cannot be applied. Second, there are – relatively few – cases where both the distant central R&D laboratory and the local R&D unit are represented in the list of authors. Hence, the outcome of the tradeoff in these cases is inconclusive. The occurrence (4% of collaborations) is not frequent enough to allow meaningful analysis as a separate collaboration category. Third, there are cases for which the central laboratory is not the local R&D unit but still in the country of the foreign university. Since here the central alternative cannot be really considered distant collaboration and features no non-spatial distance, we do not consider this category in our sample for analysis. Fourth, there is a very minor (56 observations) sample attrition due to the use of firm fixed effects in the empirical models. Some firms with a small set of foreign university collaborations invariably choose for a local R&D or a central R&D laboratory approach. In the model with firm fixed effects, these observations cannot be included because the outcome of the model is perfectly correlated with the fixed effect.

After excluding these four categories, the sample for analysis covers 12,808 observations on foreign university collaborations. These collaborations relate to 49 firms, as in particular smaller (biotech) firms tend not to have local R&D options or well-identified central laboratories, or show no variation in the choice for local versus distant collaborations. The last line in Table 2 shows the share of local collaboration for the set of focal collaborations. The share of local collaboration is declining from about 51% in 1995–2000 to 28% in 2011–2015, suggesting a declining role of proximity advantages.

The steps to arrive at the final sample for analysis ensure a parsimonious test of decisions involving tradeoffs between local and distant collaboration, but we note they imply a relatively strong sample attrition. In a supplementary analysis, we therefore examine the generality and robustness of our findings. First, we maintain collaborations where the firm has no local R&D, which nearly triples the number of observations to almost 33,000. Second, we broaden the definition of local R&D unit and local collaboration to include R&D units that are located outside the territorial grid 2–level 2 region of the university but still in a region within a 200-km radius of the university, which amounts to an increase to almost 23,000 observations.

### Focal Variables

The *dependent variable* (choice for distant rather than local collaboration) takes the value 1 if the research collaboration with a foreign university involves a central R&D unit and zero if the local R&D unit is involved. The independent variables can vary across firm *f*, research domain *d* and location *l*, or combinations thereof. In order to highlight the sources of variation we add subscripts *f, d, l* when we introduce each variable. In the case of the *rivalry* variable, the subscript *l* refers to the location of the local unit. In other instances, it is a relative measure relating to both the central and local R&D units.

The importance of *scale in research capabilities *_*(f,d,l)*_ is measured by the number of publications of the R&D units in the research domain. This is based on the idea that firms will operate larger laboratories if a domain is characterized by large-scale economies (Belderbos et al., 2013). Following prior work (Ahuja & Lampert, 2001), we use a 4-year window to measure the knowledge base of R&D units. The publications of the central laboratory in the past 4 years in the domain are divided by the number of publications of the local R&D unit in the domain in the same period, to arrive at a relative measure. Hypothesis 1 predicts a positive influence on distant collaboration. In case the local R&D unit has no prior publication activity in the particular research domain of the focal collaboration, the relative scale variable is not defined; for these observations, we include the dummy variable ‘*local laboratory not active in (basic) research domain’* and we set the value of *scale in research capabilities* to zero.

Opportunities to benefit from *knowledge diversity*_*(f,d,l)*_ in a research collaboration are measured on the basis of the relative research diversification of the central R&D unit relative to the local R&D unit. More specifically, we calculate the spread of the central R&D unit’s publications over the 42 research domains for the 4-year past publication portfolio, using the inverse of the Herfindahl index. We do the same for the local R&D unit and use the inverse Herfindahl of the local unit as the denominator. The knowledge diversity variable takes larger values when the research portfolio of the central R&D unit is more diversified than that the one from the local unit. Hypothesis 2 predicts a positive influence.

Whether the collaboration with the university focuses on basic research or applied research is assessed by making use of the CHI journal classification scheme, which assigns scientific journals to one of four levels, from applied to basic research (Hamilton, 2003). For biomedical journals, the four research levels are *clinical observation* (level 1), *clinical mix* (level 2), *clinical investigation* (level 3), and *basic biomedical research* (level 4). In line with previous research, publications in level-4 journals are considered as reporting on basic scientific research (e.g., Thursby & Thursby, 2011; Della Malva *et al.*, 2015). *Capabilities in basic research*_(f,d,l)_ is measured as the ratio of basic research publications of the central R&D laboratory over the basic research publications of the local R&D unit in the prior 4-year window. Hypothesis 3 predicts a positive influence of capabilities in basic research if the focal collaboration focuses on basic research. Capabilities in basic research is interacted with the basic research indicator for the focal collaboration, and we report separate coefficients for capabilities in basic research for collaborations in basic and applied research. As the CHI journal classification scheme has not been fully updated in recent years, we are not able to classify all collaborations as basic or applied. To avoid further sample attrition, the focal variables are calculated for those observations for which the categorization is available, while including a dummy variable ‘*basic research classification not available’* for those observations (21%) lacking this information. We control for the cases in which the local R&D unit has no prior basic research activity by including the dummy variable ‘*local laboratory not active in (basic) research domain’*.

In order to measure the *maturity*_*(d)*_ of the research domain of the collaboration, we used information from the database of Mishra and Torvik (2016) on the age of individual (detailed) MeSH terms listed on focal co-publications. The age of a MeSH term is calculated as the number of years between the year of the focal publication and the year of first publication in the PubMed database on which the particular MeSH term was recorded. The maturity of the research domain of a collaboration is measured by the age of the most recent detailed MeSH term of the co-publication. Hypothesis 4 predicts a positive influence.

We follow Patel and Pavitt (1997) and use two criteria to determine whether the collaboration concerns one of the firm’s core research domains: the domain should be important for the firm, and the firm should have relative strength in the domain. The first criterion we measure by the share of firm publications in the domain in the firm’s total publications. The second criterion is operationalized as the firm’s revealed technological advantage (RTA) in the research domain. The RTA is defined as the ratio of the firm’s share of worldwide publications in the research domain divided by the firm’s share of worldwide publications in all domains. The index has values between 0 and ∞, with values greater than 1 indicating that the firm has a revealed comparative advantage in the domain. Research domains scoring high on both criteria – defined as equal to or above the 75th percentile – are classified as core research domains of the firm. We again used a 4-year moving window for the publication variables. A research collaboration is considered to take place in a *core research domain*_*(f,d)*_ of a firm if at least one of the three-digit MeSH descriptors listed on the co-publication is a core research domain of the firm. Hypothesis 5 predicts a positive influence on distant collaboration with the central R&D unit.

The presence of *rival firms*_*(d,l)*_ is assessed by identifying publications of other firms in the same host region and in the same research domain(s) as the focal research collaboration. The intensity of rivalry in the region is measured as the number of rival firms active in the research domain of the focal co-publication in the four years prior to the collaborative research with the foreign university. We demeaned this variable in the analysis, such that the estimate of *core research domain* is evaluated at a meaningful (mean) value of the number of rivals, rather than at zero rivals. We use the publication activities of all 148 biopharmaceutical firms in our sample to determine the presence of rival firms. Hypothesis 6 predicts a positive interaction term between *core research domain* and *local rivals*.

### Control Variables

The decision to collaborate locally or at a distance may also be driven by the *local embeddedness*_*(f,d,l)*_ of the firm’s R&D unit in the host region, as embeddedness and experience in operating in local research networks are likely to make local university collaboration more attractive. The local embeddedness is measured as the ratio of the number of publications of the local R&D unit with co-authors located in the host region to the total number of publications of that unit, during the 4 years prior to the focal collaboration.

The analysis also controls for local contextual factors. We control for the *host region specialization*_*(d,l)*_ in the research domain of the collaboration. Using regional publication counts from PubMed, we calculated the revealed comparative advantage (RCA) of a region as the share of publications of the host region in the research domain of the co-publication relative to the world output in the same research domain, divided by the world share of the host region in publications across all research domains. We calculated host region specialization using a 4-year lagged window relative to the focal co-publication year. Similarly, the analysis controls for the specialization of the country of the central laboratory. The variable *central laboratory country specialization*_*(d,l)*_ in the research domain of the collaboration is the revealed comparative advantage (RCA) of the country of the central laboratory, calculated as the world share of the country in the research domain of the co-publication, divided by its world share across all research domains.

The literature has pointed out the importance of geographic and contextual distance in potentially hampering effective collaboration (e.g., Laursen et al., 2011). We therefore include measures of *geographic distance*_*(f,l)*_ and *non-spatial distance*_*(f,l)*_ between the foreign university and the central R&D laboratory. Geographic distance is measured as Euclidean distance (in kilometers, in natural logarithm) using the latitude and longitude of addresses of the foreign university and the central R&D unit. Non-spatial or “contextual” distance is measured as the principal component of cultural distance, institutional distance and language distance between the country of the foreign university and the country of the central R&D unit (e.g., Beugelsdijk et al., 2017).^10^ The measure of cultural distance draws on the six distance dimensions developed by Hofstede et al. (2010) using the aggregation method proposed by Kogut and Singh (1988). The measure of institutional distance is taken from the World Bank Worldwide Governance Indicators, for which we also use the aggregation method of Kogut and Singh (1988). To capture the language distance between the country of a firm’s central R&D unit and the foreign university we use the measure developed by Dow and Karunaratna (2006). This measure accounts for the closeness of two languages, the frequency of languages spoken and the heterogeneity of spoken languages in pairs of countries.

Although local R&D collaboration by definition implies a relatively close proximity to the foreign university in the same region, there can be heterogeneity in the distance between the local R&D unit and the foreign university, which may affect the attractiveness and probability of local collaboration. We therefore include the variable *geographic distance between the local R&D unit and the university*_*(f,l)*_, measuring the geographic distance (in natural logarithm) between the local R&D unit and the foreign university based on their longitude and latitude.

We also control for the *number of foreign universities* that are simultaneously involved in a focal research collaboration, since this may increase the benefits of coordination by the central R&D unit. Finally, we incorporate a set of *region fixed effects* capturing whether the foreign university is based in Western Europe (EU15 with Switzerland and Norway), Eastern Europe, the US or Japan a set of *country fixed effects* for the location of the central laboratory, and sets of *year fixed effects* and *firm fixed effects*. The latter control for idiosyncratic differences between firms, such as a general inclination to (de)centralize collaborative research with universities.

The descriptive statistics of the dependent and independent variables are presented in Table 3. The mean of the dependent variable indicates that distant collaboration is chosen in 62% of collaboration cases, corresponding with the information in Table 2. Central labs have on average 169 times the size of local labs in focal research domains(note that relative size variable is scaled by a factor 100) and show on average twice the level of knowledge diversity. Central labs typically have much higher basic research capabilities than local labs (a ratio of 37), while we note that 18% of collaborations focus on basic research. The average maturity of research domains is 28 years, with a large standard deviation. In 36% of the collaborations, the research domain is a core domain of the focal firm and firms are on average facing 11.4 rivals in the host region. The correlations do not indicate multicollinearity concerns.

**Table 3:** Descriptive statistics and correlation coefficients

		Mean	St. Dev.	1	2	3	4	5	6	7	8	9	10	11	12	13	14	15	16
1	Collaborative research with central R&D unit	0.62	0.49																
2	Scale in research capabilities	1.69	3.45	0.292															
3	Knowledge diversity	2.03	2.06	0.271	0.221														
4	Basic research capabilities	0.37	0.90	0.087	0.044	− 0.007													
5	Maturity	28.52	17.74	− 0.030	− 0.097	0.050	0.002												
6	Core domain	0.36	0.48	0.032	0.081	− 0.006	− 0.013	− 0.156											
7	No. of local rivals	11.43	11.89	− 0.087	− 0.164	− 0.171	− 0.018	− 0.045	− 0.036										
8	Local embeddedness	0.39	0.32	− 0.025	0.002	0.035	− 0.034	0.022	0.000	0.062									
9	Geographic distance university – local R&D	3.00	1.70	0.002	− 0.048	− 0.065	0.051	− 0.018	− 0.047	0.301	− 0.172								
10	Geographic distance university – central R&D	7.99	1.29	− 0.107	0.006	− 0.004	− 0.024	− 0.039	0.010	0.294	0.028	0.127							
11	Non-spatial distance university – central R&D	0.01	1.15	− 0.054	− 0.054	− 0.030	0.004	0.009	0.011	− 0.062	0.165	− 0.168	− 0.071						
12	Host region specialization	1.01	0.20	− 0.002	0.005	0.029	0.035	0.098	− 0.039	− 0.095	0.038	− 0.044	− 0.081	0.030					
13	Central laboratory country specialization	0.99	0.09	0.048	0.002	0.030	− 0.008	− 0.018	0.019	− 0.012	− 0.016	− 0.012	− 0.096	0.021	− 0.019				
14	No. of foreign universities in the collaboration	2.58	2.79	0.184	0.044	0.067	0.028	0.092	− 0.043	− 0.025	0.027	− 0.006	− 0.019	0.023	0.016	− 0.010			
15	Basic research collaboration	0.18	0.39	− 0.013	− 0.019	− 0.031	− 0.025	− 0.091	− 0.087	0.157	− 0.053	0.099	0.032	− 0.044	− 0.051	0.003	− 0.078		
16	Basic research indicator missing	0.20	0.40	− 0.089	− 0.034	0.002	− 0.013	0.098	− 0.025	− 0.006	0.042	− 0.050	0.008	0.040	0.009	− 0.036	0.027	− 0.238	
17	Local lab not active in (basic) research domain	0.51	0.50	0.366	0.284	0.350	− 0.353	0.033	0.053	− 0.269	0.136	− 0.223	− 0.084	0.059	0.014	0.028	0.095	− 0.113	0.046

The table includes 12,808 observations on collaborations between foreign universities and 49 focal firms. The variables relative scale (2) and relative basic research capabilities (4) are scaled by 100. Geographic distance is expressed in natural logarithm. Number of rivals and basic research capabilities are demeaned before they enter the analysis.

### Methods

As our dependent variable is binary, we employ logit models with region, firm, and year fixed effects to relate the choice between the local and headquarter R&D unit for foreign university collaboration to the focal and control variables. A Hausman test confirmed that a fixed effects model is to be preferred over a random effects model (*χ*^2^ = 194.26, *P* < 0.000).

## EMPIRICAL RESULTS

Empirical results of the fixed effect logit models are presented in Table 4. Model 1 includes only the control variables. As expected, we find that central R&D laboratories at distance are less likely to be involved in foreign university collaborations when there is a larger geographical and non-spatial distance to the host region. Further, we find that local university collaboration is more likely if the host region is specialized in the research domain of the collaboration, but that collaboration with the central laboratory is more likely the more that location is specialized in the domain. Local collaboration is less likely the larger the geographic distance between the foreign university and the local R&D unit. In contrast, collaboration through the central R&D unit is more likely if multiple university partners are involved and (once relative basic research capabilities are introduced in models 4 and 8) if the collaboration focuses on basic research. For collaborations for which a basic or applied research classification is missing, local collaboration is on average more likely. The embeddedness of the local R&D unit in local research networks is also significantly associated with local collaboration. When the local R&D unit has no prior basic research or research in the focal domain of research, collaboration with the local R&D unit is much less likely.

**Table 4:** Determinants of foreign university collaboration through a central laboratory at distance rather than through the local R&D unit

	Model 1	Model 2	Model 3	Model 4	Model 5	Model 6	Model 7	Model 8
Scale in research capabilities		0.430						0.343
		(0.000)						(0.000)
Knowledge diversity			0.527					0.339
			(0.000)					(0.000)
Basic research capabilities – basic research collaboration				1.071 (0.000)				0.864 (0.000)
Basic research capabilities – applied research collaboration				0.606 (0.000)				0.432 (0.000)
Maturity					− 0.0150			− 0.012
					(0.000)			(0.000)
Core domain						0.304	0.309	0.128
						(0.000)	(0.000)	(0.017)
No. of local rivals							− 0.0156	− 0.003
							(0.000)	(0.231)
Core domain * No. of local rivals							0.00721	0.0108
							(0.072)	(0.011)
Local embeddedness	− 0.414 (0.000)	− 0.445 (0.000)	− 0.459 (0.000)	− 0.492 (0.000)	− 0.410 (0.000)	− 0.408 (0.000)	− 0.315 (0.000)	− 0.553 (0.000)
Geographic distance university – local R&D	0.142 (0.000)	0.151 (0.000)	0.146 (0.000)	0.138 (0.000)	0.150 (0.000)	0.142 (0.000)	0.159 (0.000)	0.156 (0.000)
Geographic distance university – central R&D	− 0.271 (0.000)	− 0.302 (0.000)	− 0.276 (0.000)	− 0.243 (0.000)	− 0.270 (0.000)	− 0.273 (0.000)	− 0.241 (0.000)	− 0.263 (0.000)
Non-spatial distance university – central R&D	− 0.138 (0.000)	− 0.124 (0.000)	− 0.108 (0.000)	− 0.161 (0.000)	− 0.140 (0.000)	− 0.139 (0.000)	− 0.141 (0.000)	− 0.126 (0.000)
Host region specialization	− 0.890 (0.000)	− 0.755 (0.000)	− 0.919 (0.000)	− 0.943 (0.000)	− 0.809 (0.000)	− 0.852 (0.000)	− 0.880 (0.000)	− 0.739 (0.000)
Central laboratory country specialization	0.819 (0.002)	0.676 (0.009)	0.713 (0.007)	0.817 (0.002)	0.685 (0.008)	0.860 (0.001)	0.881 (0.001)	0.473 (0.076)
No. of foreign universities in the collaboration	0.166 (0.000)	0.156 (0.000)	0.167 (0.000)	0.158 (0.000)	0.166 (0.000)	0.168 (0.000)	0.165 (0.000)	0.158 (0.000)
Basic research collaboration	0.122 (0.040)	0.121 (0.048)	0.101 (0.096)	0.265 (0.000)	0.110 (0.065)	0.158 (0.008)	0.207 (0.001)	0.228 (0.001)
Basic research indicator missing	− 0.746 (0.000)	− 0.731 (0.000)	− 0.751 (0.000)	− 0.782 (0.000)	− 0.718 (0.000)	− 0.730 (0.000)	− 0.722 (0.000)	− 0.726 (0.000)
Local lab not active in (basic) research domain	1.910 (0.000)	1.491 (0.000)	1.481 (0.000)	2.371 (0.000)	1.933 (0.000)	1.910 (0.000)	1.864 (0.000)	1.541 (0.000)
Firm fixed effects	Included	Included	Included	Included	Included	Included	Included	Included
University region fixed effects	Included	Included	Included	Included	Included	Included	Included	Included
Central laboratory country fixed effects	Included	Included	Included	Included	Included	Included	Included	Included
Year dummies	Included	Included	Included	Included	Included	Included	Included	Included
Observations	12808	12808	12808	12808	12808	12808	12808	12808
No. of firms	49	49	49	49	49	49	49	49
Log-likelihood	− 6364.6	− 5953.0	− 6102.9	− 6107.3	− 6301.2	− 6345.3	− 6326.7	− 5623.0
(*χ*^2^) Improvement model fit (vs. Model 1)		823.3	523.4	514.7	126.8	38.66	75.75	1483.3
*P* value		(0.000)	(0.000)	(0.000)	(0.000)	(0.000)	(0.000)	(0.000)
(*χ*^2^) Overall model fit	4272.6	5095.8	4795.9	4787.2	4399.4	4311.2	4348.3	5755.9
*P* value	(0.000)	(0.000)	(0.000)	(0.000)	(0.000)	(0.000)	(0.000)	(0.000)
AUC	0.82	0.85	0.84	0.84	0.83	0.82	0.83	0.87
AIC	12909.2	12087.9	12387.9	12398.5	12784.4	12872.6	12839.5	11441.9

Results of logit models. *P* value within parentheses. AUC is the area under the curve indicator of the predictive power of the model.

The results of models 2 and 3 show that firms are more likely to collaborate via their central R&D unit when there is relatively more potential to exploit economies of scale ($\beta =0.430,$ *P* < 0.000) and knowledge diversity ($\beta =0.527, P$ < 0.000), confirming Hypotheses 1 and 2, respectively. The results of model 4 show that firms are more likely to collaborate via the central R&D unit for research that is basic in nature, the greater the relative capabilities of the central laboratory in basic research ($\beta =1.071,$ *P* < 0.000), in line with Hypothesis 3. We do find, however, that this is a wider phenomenon that is also relevant if the focal collaboration focuses on applied research: the relative basic research capability variable is also significant and positive ($\beta =0.606,$ *P* < 0.000) for applied research collaborations, though with a smaller coefficient. We confirm with a Wald test that the coefficients are significantly different in model 4 (*χ*^2^ = 16.5,* P* < 0.000) as well as in model 8 (*χ*^2^ = 14.5, *P* < 0.000). When in model 5 the maturity variable is added, its coefficient is negative ($\beta = -0.015,$ *P* < 0.000), suggesting that collaboration with the central R&D unit is more likely in the case of novel rather than mature research domains. This result implies a rejection of Hypothesis 4.

The results of model 6 show that firms are more likely to opt for centralization of collaborative R&D activities when they collaborate in their core research domains ($\beta =0.304,$ *P* < 0.000), confirming Hypothesis 5. When the interaction with the presence of rival firms is added in model 7, this interaction effect is positive and marginally significant ($\beta =0.007$, *P* = 0.072). In the fully specified model 8, this significance level increased to *P* = 0.011, in support of Hypothesis 6. The estimates imply that at the sample mean of the number of local rivals, collaboration in the core domains of the firm increases the probability of distant collaboration with the central R&D unit, and that this effect increases for high levels of rivalry. The main effect of local rivals is negative ($\beta = -0.015, P<$0.000) in model 7 but not significant in the fully specified model 8. This suggests that there is no appreciable effect of local rivals when collaboration take places in a firms’ non-core domains. If all hypothesis testing variables are included simultaneously in model 8, similar support for the hypotheses is found. The Akaike statistics (AIC) suggest that model 8 best fits the data and the incremental Wald statistics show that the hypothesis testing models are all improvements upon the controls - only model (*P* < 0.000). The AUC statistic, an indicator of how well the model predicts actual choices, also performs best in model 8. A score of 0.87 is generally regarded as a good fit.^11^

The effects of the hypothesis-testing variables on the odds ratio that distant collaboration is chosen above local collaboration are of considerable magnitude. For instance, an increase of one standard deviation in relative scale advantages in model 8 more than triples the odds that a firm collaborates with a foreign university through the central R&D unit rather than through its local R&D unit. For knowledge diversity advantages, and for relative basic research capabilities of the central laboratory (when the university collaboration focuses on basic research), the odds double. These large effects are partially explained by the relatively large standard deviations of these variables resulting from a wide variation in the resources available in local R&D units. The odds ratio increases by 14% when the collaboration involves a core research domain at the mean of local rivalry, and by 28% for a higher level of rivalry, i.e., a standard deviation above the mean. A standard deviation increase in maturity decreases the odds by about 20%.

These magnitudes of the effects of the focal hypothesis-testing variables tend to be larger than the comparable magnitude of the proximity variables driving local collaboration. The odds of distant collaboration is reduced by 14% due to a standard deviation increase in non-spatial distance, while local embeddedness reduces the odds ratio by 16% and regional specialization by 14%. The implied role of geographic distance from the central laboratory and local laboratory, is more pronounced, suggesting a 29% increase and 30% reduction in these odds, respectively.

**Figure 1:**
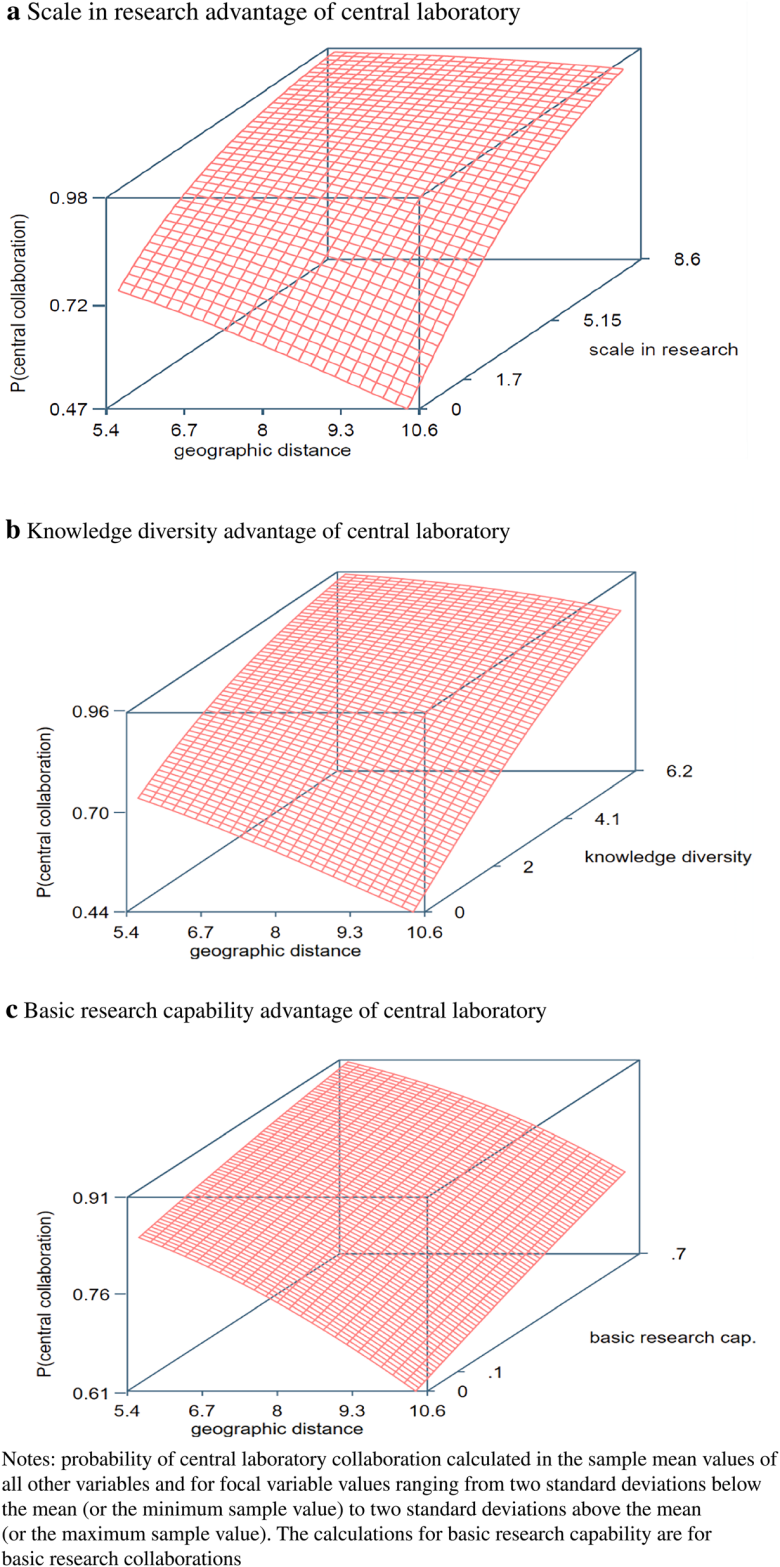
**a**–**c** Tradeoffs between proximity advantages of local collaboration and the scale in research, knowledge diversity and basic research capability advantages of central laboratory collaboration

In order to illustrate some of the tradeoffs between proximity advantages and central collaboration advantages, Figure 1a–c presents the combined effects of geographic proximity advantage of local collaboration and the scale, knowledge diversity, and basic research advantages of central collaboration at distance. The surfaces show the probability of central laboratory collaboration in the mean of all other variables, for relevant ranges of the two focal variables: two standard deviations around the mean. Figure 1a shows that at the minimum relative scale of the central laboratory, the probability of central laboratory collaboration reduces from about 74–47% when the distance with the central laboratory increases from two standard deviations below to two standard deviations above the mean. Yet, this proximity advantage of local collaboration is already fully outweighed by the scale disadvantage at around the mean of the relative scale advantage of the central laboratory (1.7). Similar patterns are observed for the knowledge diversity advantage and basic research advantages (for basic research collaboration) of the central laboratory (Figure 1b, c). These patterns show that the tradeoff between proximity advantages and the advantage of distant central laboratory collaboration can often result in collaboration at distance.

### Supplementary Analyses

We conducted a number of robustness tests, the results of which are relegated to an appendix. First, we drop the condition that the focal firm needs to have existing local R&D operations to have a credible option to decide on local R&D collaboration with the foreign university. While there are few cases (about 3%) where under such circumstances local R&D collaboration occurs, it allows the inclusion of a much larger number of observations (32,647) and a larger number of firms (82). We add a small number (one) to the denominator and denominator of the relative variables to avoid division by zero for collaboration cases without prior R&D in a local laboratory. We augment the model with a dummy variable taking the value 1 in such cases, as the distance between the local R&D unit and the university is not defined. Results closely mimic the results in Table 4. A minor exception is the effect of core domain, which now is insignificant at the sample mean of local rivals but becomes significant at a higher level of rivalry (mean plus a standard deviation).

Second, we examine the generality of our findings when enlarging the sample in another manner: by defining (collaboration with) the local R&D unit as collaboration with a R&D unit in any region within a 200-km radius of the university. This effectively brings in a large number of collaborations, by relaxing the restriction on the definition of local collaboration. The sample nearly doubles to 22,664 collaborations. The focal variables are adjusted to reflect the broader definition of local R&D. Results confirm all hypotheses.

Third, to mitigate the concern that our co-publication variables may partly pick up scientists’ mobility between universities and firms (which we are not able to measure with the data at hand) rather than collaborative research, we estimated models removing all co-publications that report more affiliations than authors. This test is based on the logic that scientists who recently transferred from academia to industry (or vice versa) may report both their new and former affiliation (although our interviews did not suggest that this is common practice). Empirical results remain robust except for a larger standard error for the core domain–local rivals interaction.

Fourth, we added a dummy variable taking the value 1 if information in the MeSH terms suggested that the research collaboration involved clinical trials. Such collaborations may involve various coordinated interactions with academic medical centers to obtain sufficient numbers of patients, with potential effects on the collaboration beyond our theoretical framework. However, the variable was insignificant, while our core results were left unchanged. Finally, we explored whether the unexpected results of maturity relate to the definition focusing on the youngest MeSH descriptor on the co-publication. Substituting the average age of the MeSH descriptor rather than the minimum did not alter results appreciably.

## DISCUSSION AND CONCLUSION

Building on the knowledge-based theory of the firm, our study aimed to uncover the antecedents of firms’ decisions to carry out collaborative research with foreign universities through a central R&D unit at distance as opposed to their local R&D unit in the university host region. The focus on the phenomenon of R&D collaborations with foreign universities in those instances where the firm has a nearby R&D affiliate provides a clear-cut setting for studying the decision of how to collaborate with universities as crucial external partners in science-intensive industries. The goal of our analysis was to obtain systematic insights into the corporate decision-making process beyond a geographic proximity-based rationale, by developing theory on factors favoring collaboration at distance related to R&D organization, knowledge appropriation and competition, and the characteristics of collaborative research in the specific research domains.

Our analysis of foreign university collaboration in the biopharmaceutical industry revealed some interesting patterns. First, despite a greater importance of central laboratories in countries outside the home country of the firms and a general increasing presence of local R&D units, the share of foreign university collaboration through a local unit as an alternative to distant collaboration with a central R&D unit has only declined over the years 1995–2015. Second, while we confirm that advantages of local collaboration are present in case of non-spatial and geographic distance between the firm’s central R&D unit and the local university, we show that these advantages can be outweighed by considerations related to the creation, transfer, recombination, and appropriation of knowledge resources. Not only do our findings indicate that firms take into account factors related to the organization of R&D, such as the relative advantage of scale, scope and basic research in central laboratories, they also highlight that the decision on how to conduct R&D with foreign universities is strategic in nature and takes into account competitive considerations. In particular, our results show that the extent to which the firm has built up knowledge and expertise in a given research domain, and the risk of this knowledge leaking to rivals in the vicinity of the foreign university, is an important concern when deciding how to carry out collaborative R&D. The implied magnitudes of the estimated effects suggest that scale, knowledge diversity and basic research capability advantages of collaboration with the central laboratory at distance easily trump the advantages of proximity.

Our analysis did not confirm the hypothesis that involvement of the central R&D unit in foreign university collaboration is more likely in mature than in novel research domains. Rather, we found the opposite, i.e., that collaborative research in relatively novel domains is more likely to involve the central laboratory. A tentative interpretation for this finding is that, while local R&D units may be in a better position to learn from local communities of practices in novel research domains, there may also be counteracting forces at play. Central R&D rather than the local R&D units may be better able to identify promising new research domains in light of the firm’s existing R&D portfolio, cross-fertilization benefits, and needs across the network of R&D facilities. If research in novel domains takes more time to bear fruit but holds the promise of breakthrough discoveries and important future growth opportunities, the central unit may also be better placed to take the lead. We suggest the role of novelty in collaborative research as a promising avenue for detailed attention in future research.

Our paper contributes to three related streams of literature. First and foremost, our research informs the literature on the internationalization of R&D in MNCs (e.g., Song & Shin, 2008; Penner, Hahn & Shaver, 2005; Belderbos et al., 2013; Cantwell & Mudambi, 2005; Lahiri, 2010; Castellani et al., 2013; Ambos & Ambos, 2011; Belderbos, 2003; Castellani & Lavoratori, 2020; Belderbos et al., 2020) by providing evidence on the different roles of local R&D units versus central R&D units in the context of scientific research endeavors of MNCs, and the related allocation of collaborative research projects. We respond to the call of a recent review on the internationalization of R&D (Papanatasiou et al., 2019) to take a broader, multidisciplinary perspective, by drawing on and integrating notions from the literatures on R&D organization and industry–science linkages. We believe that the implications of our findings extend beyond the specific question analyzed in this paper. The essence of our contribution is in demonstrating that a MNC’s decision on which units of its R&D network to mobilize for a specific R&D project depends on multiple and partly interdependent factors. A thorough understanding of how firms strategically use their R&D assets cannot draw on a single theoretical perspective but requires a more comprehensive view, in which location and distance play an important role (Beugelsdijk et al., 2010).

We show that while there is clear evidence of a greater dispersion or R&D capabilities and responsibilities abroad, the greater role of central R&D laboratories abroad does not necessarily imply an increase in local university collaboration. Rather, a tradeoff between collaboration in proximity and at distance remains and collaboration is often managed by the central R&D unit located elsewhere. We confirm prior findings that MNCs organize to take into account the risk of knowledge spillovers associated with operating foreign R&D units (Berry, 2017; Nandkumar & Srikanth, 2016), and show that in core areas of strength MNCs prefer headquarter linkages above local collaboration if the local environment is characterized by collocated rivals. Given that R&D projects in collaboration with universities may bear a greater risk of knowledge dissipation due to the open science orientation of university scientists, suggested strategies such as internal linkages between the MNC’s R&D units (Alcacer & Zhao, 2012) may be less effective for knowledge protection, such that direct control through the involvement of the central R&D unit is chosen.

Second, we complement prior research on R&D strategy and organization (e.g., Argyres & Silverman, 2004; Argyres et al., 2020; Arora et al., 2011; Chacar & Lieberman, 2003; Henderson & Cockburn, 1996; Leiponen & Helfat, 2011) by showing how scale, scope and knowledge diversity, the nature of research activities, and appropriation conditions affect R&D allocation decisions in the specific context of collaboration with foreign university scientists. In particular, our results extend previous insights on task allocation between central and localized R&D (Arora et al. 2011), by highlighting that the advantages of centralization (Leiponen & Helfat, 2011) depend crucially on the characteristics of the research project.

Finally, we contribute to the literature on industry–science linkages (e.g., Bercovitz & Feldman, 2007; Bruneel et al., 2010; Laursen et al. 2011; Mindruta, 2013; Sauermann & Stephan, 2013; Stephan & Audretsch, 1996; Subramanian et al., 2013) by qualifying the common argumentation in favor of localized knowledge spillovers. While our analysis confirms the notion in prior research that geographic and contextual distance as well as regional specialization favor collaboration in proximity, we show a set of important motivations for MNCs to reach out to foreign universities from their central R&D laboratory rather than through a local R&D unit.

While it is tempting to draw managerial lessons from the findings, our analysis did not investigate the performance consequences of the different modes of university collaboration, such that we should be cautious in suggesting managerial implications. Our analysis suggests that MNCs should carefully weigh proximity advantages of local R&D unit collaboration against the knowledge resources and control advantages of collaboration through the central laboratory. Prior work has suggested that a firm’s decisions on how to source scientific knowledge matter because they affect the firm’s capacity to build on its scientific research in technology development, and ultimately its financial performance (e.g., Arora et al., 2011, 2014). Our findings are consistent with an important ‘orchestrator’ role of the central R&D unit in the domain and the use of global R&D committees in research domains to vet and allocate R&D projects across units. A major challenge in this regard is to give sufficient room to emergent local university collaboration and novel bottom-up initiatives by supporting information exchange across units and by supporting local R&D units if these are better placed to harness such initiatives. Even if local units are not active in the research collaboration, their involvement in local networks will make them valuable assets to serve as a bridge with the central R&D laboratory.^12^

Our findings also offer guidance to actors in the host region. For example, one relevant insight for university administrators is that the local R&D units of MNCs are not the only conduits to interact with these firms: our findings show that, all else equal, the execution of basic research projects through a central R&D unit is preferred, in particular if the central unit has strong basic research capabilities. Thus, university administrators should be thoughtful to forge relationships beyond the local R&D units of MNCs to involve remote central laboratories. The results on the potentially discouraging presence of local rivals are an important insight for regional policy makers aiming to develop thriving knowledge clusters by attracting international R&D collaborations.

Our research is not free from limitations, and we mention the most salient ones. First, our empirical study is limited to the biopharmaceutical industry. While it is likely that our findings can be extended to other science-based industries, there might be differences across industries in R&D organization. For example, compared to the biopharmaceutical industry, R&D in ICT is characterized by shorter lead times that may require direct access to the relevant knowledge base. Consequently, the role of the central R&D unit may be less prominent, and local R&D units may play a more pivotal role in ICT industries. The disadvantages of collaboration at distance may also not be equal across industries, with information on new chemical entities for drug development perhaps easier to codify than frontier research in electronics.

Second, we did not investigate the performance implications of local versus distant collaboration decisions. An interesting question for further research is whether firms allocating collaborative research responsibilities in accordance with our conceptual framework also exhibit improved performance, in terms of the scientific (scientific citations to research) and technological (citations in patents to the research) impact. Third, although we argue that one of the mechanisms through which novelty plays a role in the decision to collaborate locally or at a distance is the higher tacitness of novel knowledge, we do not have an indicator of tacitness at our disposal to corroborate this. An interesting route for further research would be the development of such a tacitness measure and to examine how local versus distant collaboration decisions are influenced by the tacitness of the knowledge involved. In addition, future research could also explore the combination of (co-)publication with patent data to further provide detail on the characteristics of R&D laboratories.

Fourth, our research focused specifically on research collaborations with foreign universities, yet firms also co-publish regularly with other firms. It would be of interest to investigate whether a similar proximity versus distance tradeoff exists if collaboration is with local firms, as for example strategic knowledge appropriation considerations on collaborating in the firm’s core research domains may become even more important. Expanding the analysis along these lines would provide a much more complete picture of the different roles of local and central R&D units in the global R&D organization of MNCs. Such research endeavors could also examine in more detail the potential heterogeneities in central laboratories in terms of tasks and autonomy, which was beyond the scope of our analysis. Finally, due to the current Covid-19 pandemic, corporate and university researchers across the globe are collaborating without the face-to-face interactions that have been deemed to be essential for effective collaboration, and have been forced to be creative using various forms of online communication. An important question is to what extent, even when restrictive measures are relaxed, this experience can fundamentally change perceptions of the value of proximity and the inclination to collaborate at distance or locally. Our data have shown that overall the relative preference for collaboration in proximity was already declining up to the year 2015, perhaps as a result of the greater ease of communication at distance. These and related questions offer ample scope for future research endeavors.

## NOTES

1. Interviewees at Johnson & Johnson explained that “the R&D organization consists of a number of main research labs that take leadership for certain disease areas” and that “each disease area is managed by a R&D leader who determines the strategy, controls the budget for internal and external research programs and determines who gets involved in research collaborations”. The case study of Pfizer in Pisano et al. (2014) also notes that each research collaboration with universities has to be approved by a Joint Steering Committee that guards “the alignment with Pfizer’s larger R&D objectives”.
2. The co-publications that these examples refer to are shown in the Appendix for reviewers.
3. We show trends in central laboratories and university collaborations for 148 biopharmaceutical firms, but for the empirical analysis we focus on the collaborations of 49 firms that face a clear and identifiable tradeoff between collaboration with the local R&D unit or with a distant central laboratory.
4. The appendix for reviewers (Table A1) lists the 42 research domains and presents a number of characteristics of collaborative research in these domains.
5. Moreover, co-publication data identify far more actual collaboration activities than other databases such as RECAP or Lexis-NEXIS (Belderbos et al., 2016, p. 41).
6. The second laboratory in terms of size is on average only half as large as the central laboratory. The sample exhibits little change in the central laboratories beyond the trend towards the growth of new central laboratories with a global mandate abroad, shown in Table 1. We rely on publications for our laboratory indicators in light of our focus on collaborative co-publications. A complementary approach can be to also examine patent information and inventor locations.
7. Specifically, in the sample period: in Belgium (Wavre: vaccines), Italy (Veneto: neuroscience), UK (East of England: pharmaceuticals; Southeast: consumer healthcare), US (North Carolina and Pennsylvania: pharmaceuticals).
8. In practice, this reduces the number of observations only by about 3%. Using a less restrictive criterion for central laboratories (50 publications over all years) does not alter the empirical results.
9. We are grateful to an anonymous referee for this suggestion. The implication for the number of observations is relatively limited: about 5% of co-publications are excluded.
10. The three non-spatial distance measures load into one factor. We do not expect that economic distance plays a substantive role in the context of firm-university research collaboration.
11. The AUC indicator shows the discriminatory power of the model by taking into account both sensitivity (the fraction of central R&D unit collaborations that are correctly classified as such) and specificity (the fraction of local collaborations that are correctly classified as such) over all different threshold levels of probability to assign a prediction as ‘correct’. The indicator ranges between 0.5 and 1, with the value one indicating full sensitivity and specificity (e.g., Hosmer & Lemeshow, 2000).
12. Such a bridging role was confirmed in our interview with R&D managers. Interviewees at Johnson & Johnson stated that “opportunities can be discovered bottom-up, but decisions on collaborations are top-down”.

## Electronic supplementary material

Below is the link to the electronic supplementary material.

Supplementary material 1 (PDF 894 kb)
