# Supplementary material for: Whither geographic proximity? Bypassing local R&D units in foreign university collaboration
Source: J Int Bus Stud. 2021 Apr 12;52(7):1302–30. doi: 10.1057/s41267-021-00413-6 (PMC8039807; doi:10.1057/s41267-021-00413-6)
Supplement: Supplementary file 1 — Supplementary material 1 (PDF 894 kb) [file 41267_2021_413_MOESM1_ESM.pdf]

Whither Geographic Proximity?

Bypassing Local R&D Units in Foreign University Collaboration

- Online Appendix -

This appendix for reviewers includes characteristics of the foreign university collaboration publications per 3-digit MeSH field (Table A1), results of supplementary analyses described in the paper (Table A2), and illustration of the publication data from which the type of collaboration was derived.

In Table A1, we report the 3-digit MeSH terms and a number of indicators. The largest MeSH fields in terms of publications of our sample firms are *amino acids, peptides and proteins (D12)*, *chemical actions and uses (D27),* and *organic chemicals (D02)*. The share of basic research is relatively high in *nucleid acids, nucleotides and nucleosides (D13)*, *enzymes and coenzymes (D08)* and *macromolecular substances (D05)*. The most novel MeSH, on average, is *biological factors*, while the most mature one is *animal diseases*. There is not a great deal of variation in the share of local foreign university collaboration, with shares mostly in the 40-50 percent range.

Table A2 present empirical results for the model, with all hypothesis testing variables included, for 1) a larger sample including observations for which prior local R&D is absent; 2) a larger sample by expanding the scope of local collaboration to include R&D units of the firm in regions within a 200 kilometer range of the focal university; 3) a smaller sample after removing co-publications that report more affiliations than authors; 4) a model with an additional dummy for clinical trials included; 5) a model with the maturity variable defined as average age of the MeSh descriptors on the co-publication.

Finally, Appendix A3 contain excerpts of publication documents with author affiliations to illustrate the information used to determine local collaboration and collaboration at distance with a central laboratory.

Table A1. Indicators per MeSH 3-digit field descriptor

MeSH ID	MeSh ID field descriptor	share of publications	share basic research	share local collaboration	maturity
C01	BACTERIAL INFECTIONS AND MYCOSES	0.88	7.01	55.58	37.82
C02	VIRUS DISEASES	1.26	6.70	40.94	34.04
C03	PARASITIC DISEASES	0.23	10.68	37.86	54.00
C04	NEOPLASMS	3.08	11.54	41.12	39.13
C05	MUSCULOSKELETAL DISEASES	1.23	4.44	35.00	57.59
C06	DIGESTIVE SYSTEM DISEASES	1.40	7.94	45.71	28.82
C07	STOMATOGNATHIC DISEASES	0.12	3.70	42.59	43.07
C08	RESPIRATORY TRACT DISEASES	1.35	6.59	49.16	45.56
C09	OTORHINOLARYNGOLOGIC DISEASES	0.15	9.23	64.62	50.13
C10	NERVOUS SYSTEM DISEASES	2.07	21.48	39.65	45.05
C11	EYE DISEASES	0.23	5.83	37.86	29.52
C12	MALE UROGENITAL DISEASES	0.53	5.63	51.52	42.46
C13	FEMALE UROGENITAL DISEASES AND PREGNANCY COMPLICATIONS	0.95	6.00	44.84	37.73
C14	CARDIOVASCULAR DISEASES	2.55	7.42	41.64	40.70
C15	HEMIC AND LYMPHATIC DISEASES	1.09	5.83	36.46	37.36
C16	CONGENITAL, HEREDITARY, AND NEONATAL DISEASES AND ABNORMALITIE	0.53	12.34	30.64	31.48
C17	SKIN AND CONNECTIVE TISSUE DISEASES	1.23	5.75	37.66	47.25
C18	NUTRITIONAL AND METABOLIC DISEASES	1.62	8.58	38.96	31.67
C19	ENDOCRINE SYSTEM DISEASES	0.86	8.44	40.11	43.90
C20	IMMUNE SYSTEM DISEASES	1.53	8.48	47.92	39.61
C22	ANIMAL DISEASES	0.51	19.28	50.67	59.78
C23	PATHOLOGICAL CONDITIONS, SIGNS AND SYMPTOMS	4.83	11.60	42.22	38.04
C25	CHEMICALLY-INDUCED DISORDERS	0.34	8.00	40.67	53.30
C26	WOUNDS AND INJURIES	0.36	14.65	35.67	38.23
D01	INORGANIC CHEMICALS	2.14	28.34	53.50	50.68
D02	ORGANIC CHEMICALS	8.20	15.30	43.53	32.17
D03	HETEROCYCLIC COMPOUNDS	7.43	14.73	39.82	29.39
D04	POLYCYCLIC COMPOUNDS	2.89	13.55	46.26	30.38
D05	MACROMOLECULAR SUBSTANCES	0.77	39.12	55.88	29.81

Table A1. Indicators per MeSH 3-digit field descriptor (continued)

D06	HORMONES, HORMONE SUBSTITUTES, AND HORMONE ANTAGONISTS	1.93	16.27	42.10	22.39
D08	ENZYMES AND COENZYMES	5.23	34.94	44.13	21.46
D09	CARBOHYDRATES	2.12	19.78	45.48	32.88
D10	LIPIDS	1.89	24.16	45.67	31.98
D12	AMINO ACIDS, PEPTIDES, AND PROTEINS	14.03	26.42	40.72	20.62
D13	NUCLEIC ACIDS, NUCLEOTIDES, AND NUCLEOSIDES	3.26	35.10	45.31	27.36
D20	COMPLEX MIXTURES	1.00	10.27	49.54	29.79
D23	BIOLOGICAL FACTORS	5.60	20.00	42.72	18.58
D25	BIOMEDICAL AND DENTAL MATERIALS	0.54	13.03	54.20	37.39
D26	PHARMACEUTICAL PREPARATIONS	1.45	5.64	49.37	44.86
D27	CHEMICAL ACTIONS AND USES	12.60	13.77	42.22	33.08
	TOTAL	100			

Notes: Averages for the sample of 12808 co-publications with foreign universities of the 49 sample firms. A publication can have multiple MESH descriptors.

Table A2. Results of Alternative Specifications

	no local R&D unit obs. Included	200K local R&D definition extension	no multiple affiliation	clinical trial dummy	average maturity
Scale in research capabilities	0.272	0.341	0.359	0.343	0.524
	(0.000)	(0.000)	(0.000)	(0.000)	(0.000)
Knowledge diversity	0.164	1.080	0.345	0.338	0.122
	(0.000)	(0.000)	(0.000)	(0.000)	(0.000)
Basic research capabilities - basic research collaboration	0.854	1.078	0.880	0.864	0.852
	(0.000)	(0.000)	(0.000)	(0.000)	(0.000)
Basic research capabilities - applied search collaboration	-0.0492	0.413	0.409	0.432	0.367
	(0.110)	(0.000)	(0.000)	(0.000)	(0.000)
Maturity	-0.00787	-0.0101	-0.0132	-0.0129	-0.0178
	(0.000)	(0.000)	(0.000)	(0.000)	(0.000)
Core Domain	0.0530	0.125	0.0925	0.130	0.257
	(0.304)	(0.001)	(0.129)	(0.015)	(0.000)
#Local Rivals	-0.0193	0.00776	-0.00490	-0.00332	0.00201
	(0.452)	(0.001)	(0.125)	(0.232)	(0.478)
Core Domain * # Local Rivals	0.0107	0.00665	0.00640	0.0107	0.00930
	(0.005)	(0.069)	(0.177)	(0.012)	(0.030)
Local Embeddedness	-0.534	-0.568	-0.448	-0.553	-0.581
	(0.000)	(0.000)	(0.000)	(0.000)	(0.000)
Geographic distance univeristy - local R&D	0.0573	0.0859	0.146	0.156	0.148
	(0.000)	(0.000)	(0.000)	(0.000)	(0.000)
Geographic distance univeristy - central R&D	-0.372	-0.396	-0.257	-0.263	-0.272
	(0.000)	(0.000)	(0.000)	(0.000)	(0.000)
Non-spatial distance university - central R&D	-0.177	-0.161	-0.137	-0.125	-0.116
	(0.000)	(0.000)	(0.000)	(0.000)	(0.000)
Host region specialization	0.154	-0.0470	-0.750	-0.736	-0.791
	(0.330)	(0.799)	(0.000)	(0.000)	(0.000)
Central laboratory country specialization	-0.587	-0.328	0.526	0.472	0.162
	(0.000)	(0.004)	(0.085)	(0.076)	(0.000)
# foreign universities in the collaboration	0.159	0.104	0.132	0.158	0.147
	(0.000)	(0.000)	(0.000)	(0.000)	(0.032)
Basic Research collaboration	1.044	0.365	0.280	0.220	-0.743
	(0.000)	(0.000)	(0.000)	(0.001)	(0.000)
Basic research indicator missing	-0.717	-0.506	-0.687	-0.728	1.136
	(0.000)	(0.000)	(0.000)	(0.000)	(0.000)
Local lab not active in (basic) research domain	1.321	1.490	1.648	1.544	
	(0.000)	(0.000)	(0.000)	(0.000)	
Clinical trial				-0.102	
				(0.366)	
geographic distance local university missing	0.353				
	(0.211)				
Firm/year/university region/CL country FE	Included	Included	Included	Included	Included
Observations	32647	22664	9947	12808	12808
N Firms	82	49	46	49	49
Log-Likelihood	-7265.4	-11048.6	-4323.6	-5622.5	-5515.2
(χ2) Improvement model fit (vs Model 1)	1384.8	2133.2	1148.9	1482.6	1708.9
P value	(0.000)	(0.000)	(0.000)	(0.000)	(0.000)
(χ2) Overall model fit	16494.2	8695.7	4588	5756.7	5971.5
P value	(0.000)	(0.000)	(0.000)	(0.000)	(0.000)
AUC	0.94	0.84	0.87	0.87	0.88
AIC	14742.9	22291.3	8837.3	11443.1	11226.3

Notes: Results of logit models. P-value within parentheses.

Appendix A3. Examples of local and distant collaboration

Examples of distant collaboration through a central lab

Example 1: Co-publication of Abbott Labs involving its central lab in Illinois (USA) and the University of Oxford and Imperial College London (UK) in the journal *AIDS* in 2001. Not involved in the co-publication was Abbott's local R&D unit in London.

Comparison of the effectiveness of non-nucleoside reverse transcriptase inhibitor-containing and protease inhibitor-containing regimens using observational databases

By: Ghani, AC (Ghani, AC); Henley, WE (Henley, WE); Donnelly, CA (Donnelly, CA); Mayer, S (Mayer, S); Anderson, RM (Anderson, RM)

AIDS
Volume: 15 Issue: 9 Pages: 1133-1142
DOI: 10.1097/00002030-200106150-00008
Published: JUN 15 2001
View Journal Information

Abstract

Objectives: To compare the effectiveness of first protease inhibitor (PI)-containing and non-nucleoside reverse transcriptase inhibitor (NNRTI)-containing regimens.

Methods: Data were analysed from three large HIV patient databases: Apache HIV Insight (TM) (APACHE), Target Management Services (TMS) and Clinical Partners (CP). The effectiveness of therapy was the time taken for HIV-1 RNA to fall below detectable levels on first highly active antiretroviral therapy regimen (PI- or NNRTI-containing) and the subsequent time to failure (two consecutive detectable measurements). Comparisons were made using proportional hazards models, adjusting for differences in age, sex, previous reverse transcriptase inhibitor use, calendar year and baseline viral load and CD4 T-cell count.

Results: The type of regimen was not associated with time to undetectable viral load in any of the three databases, all of which had high power to detect a difference. PI-containing regimens were significantly less likely to fail after reaching undetectable viral loads for APACHE and CP patients (relative hazard, 1.7; 95% confidence interval, 1.3-2.1 and relative hazard, 1.6; 95% confidence interval, 1.0-2.5 respectively). These results remained significant after allowing for an unmeasured confounder with moderate effect on risk. No significant association between time to failure and regimen was found for TMS patients, possibly due to low power (67% to detect a relative hazard of 1.5). No difference was found between regimens in the time taken for an increase of > 100 X 10(9) cells/l in CD4 T-cell count. In the APACHE database, those on NNRTI-containing regimens were more likely to have a failing CD4 T-cell response.

Conclusions: PI-containing regimens have a lower risk of treatment failure than NNRTI-containing regimens. (C) 2001 Lippincott Williams & Wilkins.

Keywords

Author Keywords: HIV-1; AIDS; antiretroviral therapy; protease inhibitors; non-nucleoside reverse transcriptase inhibitors; observational databases

KeyWords Plus: VIRUS TYPE-1 RNA; PLASMA HIV RNA; ANTIRETROVIRAL THERAPY; CLINICAL-TRIALS; LYMPHOCYTE COUNTS; RANDOMIZED TRIALS; CUBIC MILLIMETER; PLUS INDINAVIR; INFECTION; EFFICACY

Author Information

Reprint Address: Ghani, AC (reprint author)

+ Univ London Imperial Coll Sci Technol & Med, Sch Med, Dept Infect Dis Epidemiol, Norfolk Pl, London W2 1PG, England.

Addresses:

+ [1] Univ London Imperial Coll Sci Technol & Med, Sch Med, Dept Infect Dis Epidemiol, London W2 1PG, England
+ [2] Univ Oxford, Wellcome Trust Ctr Epidemiol Infect Dis, Oxford OX1 2JD, England
+ [3] Abbott Labs, Abbott Pk, IL 60064 USA

Example 2: Co-publication of Pfizer involving its central lab in Groton (Connecticut, USA) and the University of Cambridge (UK) in the journal *Organic and Biomolecular Chemistry* in 2015. Not involved in the co-publication were Pfizer's local R&D units in Cambridge, Hurley (Berkshire) or Sandwich (Kent).

Cyclopropanation using flow-generated diazo compounds

By: Roda, NM (Roda, Nuria M.)[1]; Tran, DN (Tran, Duc N.)[1]; Battilocchio, C (Battilocchio, Claudio)[1]; Labes, R (Labes, Ricardo)[1]; Ingham, RJ (Ingham, Richard J.)[1]; Hawkins, JM (Hawkins, Joel M.)[2]; Ley, SV (Ley, Steven V.)[1]
View Web of Science ResearcherID and ORCID

ORGANIC & BIOMOLECULAR CHEMISTRY
Volume: 13 **Issue:** 9 **Pages:** 2550-2554
DOI: 10.1039/c5ob00019j
Published: 2015
Document Type: Article
View Journal Impact

Abstract
We have devised a room temperature process for the cyclopropanation of electron-poor olefins using unstabilised diazo compounds, generated under continuous flow conditions. This protocol was applied to a wide range of different diazo species to generate functionalised cyclopropanes which are valuable 3D building blocks.

Keywords
KeyWords Plus: STEREOSELECTIVE CYCLOPROPANATION; INTRAMOLECULAR CYCLOPROPANATION; MANGANESE-DIOXIDE; NATURAL-PRODUCTS; AMINO-ACIDS; OXIDATION; CHEMISTRY; REACTORS; CYCLIZATIONS; ARTEMISININ

Author Information
Reprint Address: Ley, SV (reprint author)

+ Univ Cambridge, Dept Chem, Innovat Technol Ctr, Lensfield Rd, Cambridge CB2 1EW, England.

Addresses:

+ [1] Univ Cambridge, Dept Chem, Innovat Technol Ctr, Cambridge CB2 1EW, England

+ [2] Pfizer Inc, Worldwide Res & Dev, Groton, CT 06340 USA

Examples of local collaboration

Example 1: Co-publication of AstraZeneca (headquartered in the UK) with the University of Sydney in the European Respiratory Journal in 2000, through its local R&D unit in New South Wales.

Optimal asthma control, starting with high doses sf inhaled budesonide

By: Reddel, HK (Reddel, HK); Jenkins, CR (Jenkins, CR); Marks, GB (Marks, GB); Ware, SI (Ware, SI); Xuan, W (Xuan, W); Salome, CM (Salome, CM); Badcock, CA (Badcock, CA); Woolcock, AJ (Woolcock, AJ)

EUROPEAN RESPIRATORY JOURNAL
Volume: 16 **Issue:** 2 **Pages:** 226-235
DOI: 10.1034/j.1399-3003.2000.16b08.x
Published: AUG 2000
View Journal Information

Abstract

The aim of this study was to determine whether outcomes in poorly controlled asthma can be further improved with a starting dose of inhaled budesonide higher than that recommended in international guidelines.

The study had a parallel-group design and included 61 subjects with poorly controlled asthma, randomized to receive 3,200 mu g or 1,600 mu g budesonide daily by Turbuhaler(R) for 8 weeks (double-blind), then 1,600 mu g.day(-1) for 8 weeks (single-blind), followed by 14 months of open-label budesonide dose down-titration using a novel algorithm, with a written asthma crisis plan based on electronic peak expiratory flow monitoring. The primary outcome variable for weeks 1-16 was change in airway hyperresponsiveness (AHR), and, for the open-label phase, mean daily budesonide dose.

By week 16, there were large changes from baseline in all outcomes, with no significant differences between the 3,200- and 1,600-mu g.day(-1) starting dose groups (AHR increased by 3.2 versus 3.0 doubling doses, p=0.7; morning peak flow increased by 134 versus 127 L.min(-1), p=0.8). Subjects starting with 3,200 mu g.day(-1) were 3.8 times more likely to achieve AHR within the normal range, as defined by a provocative dose of histamine causing a 20% fall in forced expiratory volume in one second (PD20) of greater than or equal to 3.92 mu mol by week 16 (p=0.3). During dose titration, there was no significant difference in mean budesonide dose (1,327 versus 1,325 mu g.day(-1), p>0.3). Optimal asthma control was achieved in the majority of subjects (at completion/withdrawal: median symptoms 0.0 days.week(-1), beta(2)-agonist use 0.2 occasions.day(-1), and PD20 2.4 mu mol).

In subjects with poorly controlled asthma, a starting dose of 1,600 mu g.day(-1) budesonide was sufficient to lead to optimal control in most subjects. The high degree of control achieved, compared with previous studies, warrants further investigation.

Keywords

Author Keywords: asthma; asthma prevention and control; bronchial hyperreactivity; budesonide; drug administration schedule; randomized controlled trials
KeyWords Plus: PEAK EXPIRATORY FLOW; LONG-TERM TREATMENT; CLINICAL ASTHMA; AIRWAY HYPERRESPONSIVENESS; BRONCHIAL RESPONSIVENESS; DEPENDENT ASTHMATICS; CONTROLLED TRIAL; MILD ASTHMA; CORTICOSTEROIDS; MANAGEMENT

Author Information

Reprint Address: Reddel, HK (reprint author)
Royal Prince Alfred Hosp, Inst Resp Med, POB M77, Camperdown, NSW 2050, Australia.
Addresses:
[1] Royal Prince Alfred Hosp, Inst Resp Med, Camperdown, NSW 2050, Australia
[2] Univ Sydney, Camperdown, NSW, Australia
[3] AstraZeneca Australia, N Ryde, NSW, Australia

Example 2: Co-publication of Sanofi Pasteur (the vaccines division of Sanofi-Aventis, headquartered in France) with Harvard University (US), the HHMI (US), the Brigham & Womens hospital (US) and Umea University (Sweden) in the journal *Infection and Immunity* in 2014, through its local R&D unit in Cambridge, US.

Differential Requirement for PBP1a and PBP1b in In Vivo and In Vitro Fitness of Vibrio cholerae

By: Dorr, T (Doerr, Tobias)[1,2,3]; Moll, A (Moell, Andrea)[1,2,3]; Chao, MC (Chao, Michael C.)[1,2,3]; Cava, F (Cava, Felipe)[4]; Lam, H (Lam, Hubert)[5]; Davis, BM (Davis, Brigid M.)[1,2,3]; Waldor, MK (Waldor, Matthew K.)[1,2,3]

View Web of Science ResearcherID and ORCID

INFECTION AND IMMUNITY

Volume: 82 **Issue:** 5 **Pages:** 2115-2124

DOI: 10.1128/IAI.00012-14

Published: MAY 2014

Document Type: Article

View Journal Impact

Abstract

We investigated the roles of the Vibrio cholerae high-molecular-weight bifunctional penicillin binding proteins, PBP1a and PBP1b, in the fitness of this enteric pathogen. Using a screen for synthetic lethality, we found that the V. cholerae PBP1a and PBP1b proteins, like their Escherichia coli homologues, are each essential in the absence of the other and in the absence of the other's putative activator, the outer membrane lipoproteins LpoA and LpoB, respectively. Comparative analyses of V. cholerae mutants suggest that PBP1a/LpoA of V. cholerae play a more prominent role in generating and/or maintaining the pathogen's cell wall than PBP1b/LpoB.V. cholerae lacking PBP1b or LpoB exhibited wild-type growth under all conditions tested. In contrast, V. cholerae lacking PBP1a or LpoA exhibited growth deficiencies in minimal medium, in the presence of deoxycholate and bile, and in competition assays with wild-type cells both in vitro and in the infant mouse small intestine. PBP1a pathway mutants are particularly impaired in stationary phase, which renders them sensitive to a product(s) present in supernatants from stationary-phase wild-type cells. The marked competitive defect of the PBP1a pathway mutants in vivo was largely absent when exponential-phase cells rather than stationary-phase cells were used to inoculate suckling mice. Thus, at least for V. cholerae PBP1a pathway mutants, the growth phase of the inoculum is a key modulator of infectivity.

Keywords

KeyWords Plus: PENICILLIN-BINDING PROTEINS; BETA-LACTAM ANTIBIOTICS; ESCHERICHIA-COLI K-12; BACTERIAL-CELL WALL; D-AMINO ACIDS; PEPTIDOGLYCAN SYNTHASES; DELETION; BIOSYNTHESIS; AFFINITIES; MOTILITY

Author Information

Reprint Address: Waldor, MK (reprint author)

+ Brigham & Womens Hosp, Div Infect Dis, 75 Francis St, Boston, MA 02115 USA.

Addresses:

+ [1] Brigham & Womens Hosp, Div Infect Dis, Boston, MA 02115 USA

+ [2] Harvard Univ, Sch Med, Dept Microbiol & Immunobiol, Boston, MA USA

+ [3] HHMI, Boston, MA USA

+ [4] Umea Univ, Dept Mol Biol, Lab Mol Infect Med Sweden, Umea, Sweden

+ [5] Sanofi Pasteur, Discovery Res, Cambridge, MA USA

Remark: We consider the collaboration of AstraZeneca with Harvard as local. This is not the case for the collaboration of AstraZeneca with Umea University in Sweden (which is treated as another observation).
